# Phylogenetic analysis of migration, differentiation, and class switching in B cells

**DOI:** 10.1371/journal.pcbi.1009885

**Published:** 2022-04-25

**Authors:** Kenneth B. Hoehn, Oliver G. Pybus, Steven H. Kleinstein

**Affiliations:** 1 Department of Pathology, Yale School of Medicine, New Haven, Connecticut, United States of America; 2 Department of Zoology, University of Oxford, Oxford, United Kingdom; 3 Department of Pathobiology and Population Sciences, Royal Veterinary College London, London, United Kingdom; 4 Program in Computational Biology and Bioinformatics, Yale University, New Haven, Connecticut, United States of America; 5 Department of Immunobiology, Yale School of Medicine, New Haven, Connecticut, United States of America; Columbia University Medical Center: Columbia University Irving Medical Center, UNITED STATES

## Abstract

B cells undergo rapid mutation and selection for antibody binding affinity when producing antibodies capable of neutralizing pathogens. This evolutionary process can be intermixed with migration between tissues, differentiation between cellular subsets, and switching between functional isotypes. B cell receptor (BCR) sequence data has the potential to elucidate important information about these processes. However, there is currently no robust, generalizable framework for making such inferences from BCR sequence data. To address this, we develop three parsimony-based summary statistics to characterize migration, differentiation, and isotype switching along B cell phylogenetic trees. We use simulations to demonstrate the effectiveness of this approach. We then use this framework to infer patterns of cellular differentiation and isotype switching from high throughput BCR sequence datasets obtained from patients in a study of HIV infection and a study of food allergy. These methods are implemented in the R package *dowser*, available at https://dowser.readthedocs.io.

## Author summary

B cells produce high affinity antibodies through an evolutionary process of mutation and selection during adaptive immune responses. Migration between tissues, differentiation to cellular subtypes, and switching between different antibody isotypes can be important factors in shaping the role B cells play in response to infection, autoimmune disease, and allergies. B cell receptor (BCR) sequence data has the potential to elucidate important information about these processes. However, there is currently no robust, generalizable framework for making such inferences from BCR sequence data. Here, we develop three parsimony-based summary statistics to characterize migration, differentiation, and isotype switching along B cell phylogenetic trees. We confirm the effectiveness of our approach using simulations and further apply our method to data from patients with HIV and allergy. Our methods are released in the R package *dowser*: https://dowser.readthedocs.io.

## Introduction

The adaptive immune system in humans depends on B cells to produce antibodies capable of neutralizing a wide array of pathogens. Antibody structures are initially expressed as B cell receptors (BCRs) on the surfaces of B cells. BCRs are generated through random V(D)J recombination and then subjected to repeated rounds of somatic hypermutation (SHM), cell proliferation, and selection for antigen binding [1]. This evolutionary process, called affinity maturation, creates many lineages of B cells that each descend from a single naive progenitor cell. Cells within a clonal lineage differ predominately by point mutations. The genetic variation within these clonal lineages has long been investigated using phylogenetic methods [2]. High throughput sequencing of BCR sequences has shown promise in elucidating information about the adaptive immune response in humans, such as the sequence of mutations that occur during antibody co-evolution with HIV [3], and the process of mutation and selection during affinity maturation generally [4]. Other important biological processes may occur as BCR sequences evolve, such as B-cell migration between tissues [5], differentiation into cellular subsets [6], and antibody isotype class switching [7]. If these processes co-occur with SHM, then in principle they can be investigated and inferred using phylogenetic techniques.

Understanding the ways in which B cells migrate between tissues, differentiate into cell types, and switch isotypes can provide important information about the mechanism and treatment of B cell mediated diseases. For instance, previous work showed that B cells found in the brain lesions of multiple sclerosis patients arise from the cervical lymph nodes, which can be more easily targeted for treatment [5]. Other work in the autoimmune disease myasthenia gravis indicates that pathogenic B cells migrate from the thymus to the peripheral blood, suggesting they may be targeted for treatment with early thymectomy [8]. In the study of food allergies, B cell repertoire analysis has been used to show that allergy-causing IgE cells likely originate from distinct memory B cell subsets [9], and from sequential switching through IgG or IgA rather than from IgM [10]. Further work has shown that IgE producing B cells likely undergo isotype switching locally in gut associated tissue [11]. All of these processes have the potential to be studied using a combination of BCR repertoire analysis and molecular phylogenetic methods, so long as SHM occurs between transition events. This SHM could occur either through germinal center reactions, or through extrafollicular activation and SHM [12]. Possible scenarios in which migration among tissues could be studied in this way are shown in **S1 Fig**.

Migration and cellular differentiation in B cells can be viewed as analogous to geographic spread of rapidly evolving viruses, the study of which–viral phylogeography–has advanced both in theory and application in the past decade (e.g. [13]). For example, phylogeographic methods have been used to determine the origin of the HIV pandemic [14], factors influencing the recent Ebola epidemic [15,16], and the epidemic spread of Zika virus [17,18]. Modern phylogeographic analyses typically model phylogenetic sequence evolution [19], and changes in geographic location within a unified framework [20,21]. Successfully developing a phylogeographic framework for B cell lineages would enable the testing of new hypotheses regarding the nature of evolution during affinity maturation.

There are serious challenges that must be addressed before using modern phylogeographic methods on B cell repertoire datasets. Such techniques typically rely on molecular clock trees, whose branch lengths represent elapsed time between nodes [20]. Accurately modeling sequence change through time requires either data sampled at multiple time points, or prior information about the expected rate of sequence evolution. These are frequently not available for B cell lineages. Data samples, particularly biopsies, are often only taken at a single time point [5,22], and the variation of B cell mutation rate over time is largely unknown and likely dependent on cell subset. Even using a Markov model to describe state changes along B cell molecular phylogenies is not straightforward: B cell lineage trees frequently contain identical sequences with different states. This results in state changes across zero-length branches, which do not fit within a Markov model framework. Further, modern phylogeographic techniques often rely on Markov chain Monte Carlo sampling, which makes them computationally intensive and impractical to apply to thousands of sequences. B cell bulk repertoires often contain millions of sequences, and individual lineages sometimes contain thousands of unique sequences.

We propose that hypotheses about B cell migration and differentiation may be usefully investigated using heuristic summary statistics that characterize the distribution of trait values along phylogenetic trees. Indeed, such heuristic approaches which do not depend on branch length have historically been used to test hypotheses about migration between populations [23–25]. While tree based summary statistics have been previously used to assess B cell migration [5], differentiation [6], and isotype switching [7], these approaches have not been tested through simulations and their general accuracy is unclear. To address this methodological gap, we develop a set of maximum parsimony-based statistics that summarize the relative distribution of B cell states along the tips of lineage trees within repertoires and introduce a framework for assessing the significance of their difference from randomized trees. We demonstrate through simulations that these tests relate intuitively to different regimes of migration and differentiation. To demonstrate its utility, we use this framework to test hypotheses regarding differentiation of cell types in HIV infection, and sequential class switching to IgE and IgG4. We introduce a statistically principled and scalable means of analyzing the evolution of discrete traits in B cell repertoires. We release these methods in the R package *dowser*.

## Methods

### Predicting states of internal tree nodes

The goal of the discrete trait analysis framework presented here is to characterize the distribution of predicted trait values along the tips of B cell lineage trees. Given an alignment of sequences inferred to descend from the same naive ancestor (i.e. the same clonal lineage), lineage tree topologies and branch lengths were estimated using maximum parsimony using *dnapars* v3.967 [26]. While other methods are available for inferring B cell lineage trees, maximum parsimony has been shown to be effective in some cases [27] and is faster than more complex maximum likelihood models designed for B cell lineages [4,28]. Importantly, the statistics presented here are not limited to tree topologies inferred through maximum parsimony; maximum parsimony is used here primarily for computational expediency.

Maximum parsimony is also used to infer the discrete character states (e.g. cell subtype, isotype, tissue) of internal nodes, given a tree in which each tip is associated with a given character state. Nodes with different states from their immediate ancestors are counted as state changes. More specifically, internal node states were reconstructed using the Sankoff dynamic programming maximum parsimony algorithm [29], which, given a weight matrix for each type of state change, determines the minimum number of state changes that must be made along the tree given the states at the tips. The backtrace step of this algorithm can be used to determine a set of most parsimonious internal node states. Often there are multiple such maximum parsimony sets. To represent state changes across ambiguous internal node sets, trajectories with equal parsimony were randomly chosen in the backtrace step of the Sankoff algorithm, beginning at the root of the tree and moving towards the tips. This process was performed 100 times for each tree, and the mean of each type of state change was reported.

Strictly bifurcating B cell lineage trees frequently have clusters of nodes separated by zero-length branches (soft polytomies), which represent a high degree of uncertainty in tree topology. This uncertainty in the order of bifurcating nodes can result in a potentially large number of uninformative state changes along the polytomy. Multiple steps were taken to minimize the effects of random polytomy resolution (**S1 Appendix**). Briefly, nodes within each polytomy were first re-ordered to minimize the number of state changes along the tree. To represent the uncertainty in the order of state changes, nodes within each polytomy were grouped together into separate subtrees according to their predicted state. These state-specific subtrees were then joined together in a balanced manner, ensuring that state changes could occur in any direction among the states contained within the polytomy **(S1 Appendix**).

### Testing trait-phylogeny association

**Fig 1:**
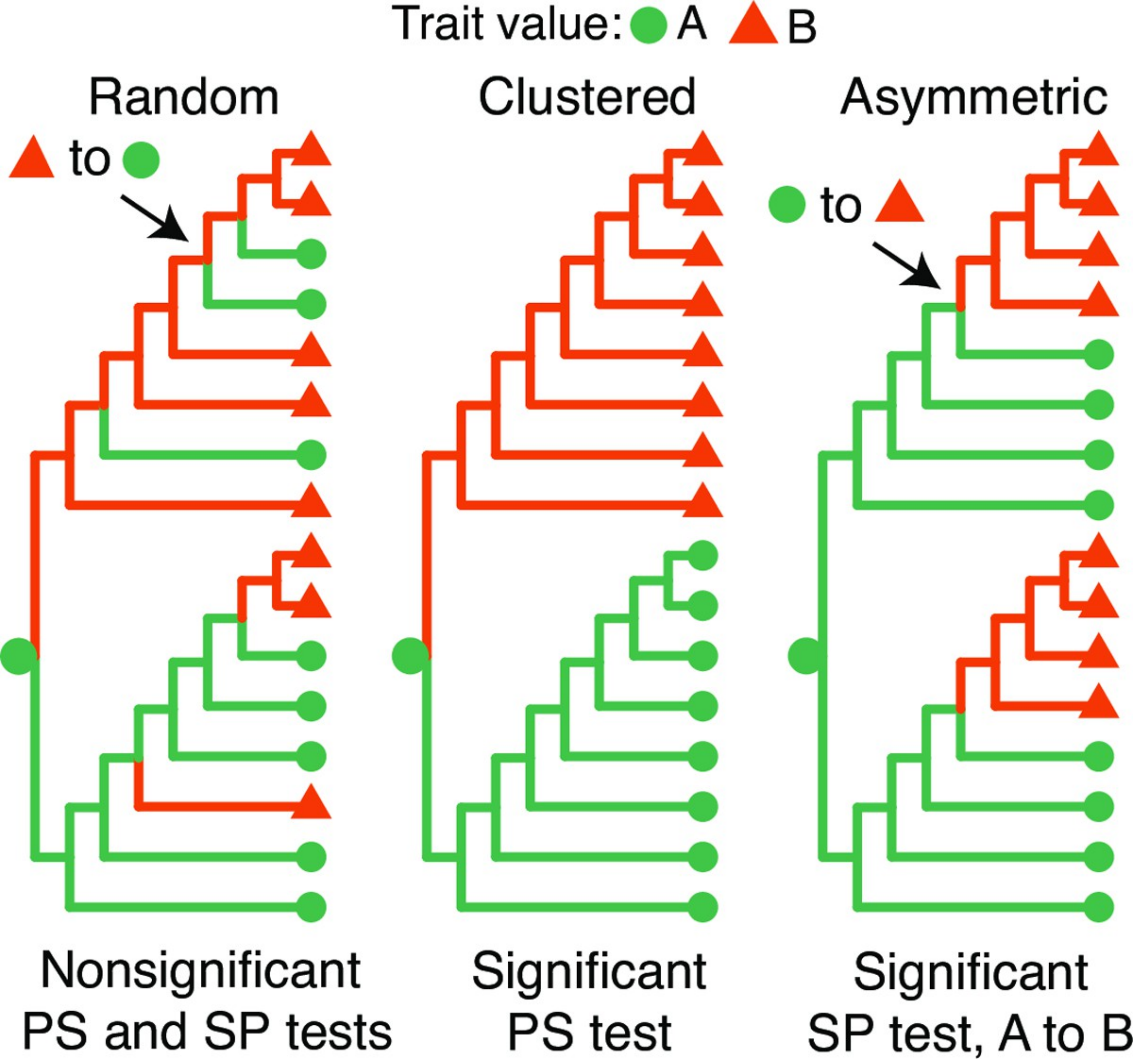
Hypothetical phylogenetic trees used to illustrate tree/trait association statistics. Trait values at internal nodes of the tree are inferred using maximum parsimony given the trait values at the tips, which are shown using different colors and shapes. Two example state changes are highlighted. **(left)** *No association between tip-trait values and tree*: Distribution of traits across this tree is indistinguishable from randomly distributed traits by any statistic used. **(middle)** *Tip-trait values clustered in tree*: Cells with the same trait value are more closely related to each other in the tree, which will yield significantly fewer switches than in the same tree with permuted tips, and therefore a significantly low *PS* statistic. **(right)** *Asymmetric ancestor/descendant relationships among trait values*: All switches in this tree are from *A* to *B* (*SP* = 1), significantly higher than expected from the same tree with permuted tips. This indicates an asymmetric relationship between these states along the tree.

Analysis begins with a B-cell lineage tree topology with discrete character states (trait values) associated with each tip, and internal node states reconstructed through maximum parsimony. The goal of our discrete trait analysis framework is to determine how the distribution of discrete character states along the internal nodes of a tree differs from its expectation if traits are randomly distributed among the tips. The statistics introduced herein are shown graphically in **Fig 1**. More formally, if there are *m* possible discrete character states, and *o*_*ij*_ is the number of state changes from type *i* to type *j*, the three statistics investigated are defined as:

$$PS=\sum_{i}^{m}\sum_{j,j\neq i}^{m}o_{ij}$$ (1)

$${SC}_{ij}=o_{ij}$$ (2)

$${SP}_{ij}=\frac{o_{ij}}{\sum_{i}^{m}\sum_{j,j\neq i}^{m}o_{ij}}$$ (3)

The *PS* (parsimony score) statistic is the total number of state changes along a tree. The *SC* (switch count) statistic from *i* to *j* is the number of state changes from state *i* to *j*. The *SP* (switch proportion) statistic from *i* to *j* is the proportion of state changes from state *i* to *j*.

We calculate the significance of these three statistics using a permutation test. This is done by randomizing traits at the tips of the lineage tree, re-calculating each statistic on the permuted tree, and repeating for a specified number of replicates. For each replicate we calculate the difference between the statistic calculated on the observed tree and the same statistic calculated on the permuted tree. We refer to this difference as δ. If mean δ > 0 (hereafter mean δ is indicated by **δ**), this indicates the statistic is on average higher in observed trees than in permuted trees. For a one-tailed test, we calculate the *p* value that **δ** > 0 as the proportion of replicates in which δ ≤ 0. Similarly, we calculate the *p* value that **δ** < 0 as the proportion of replicates in which δ ≥ 0. For a two-tailed test, we calculate the *p* value that **δ** > 0 as the proportion of replicates in which δ < 0, plus half of the replicates in which δ = 0. We refer to the calculation of these *p* values for the statistics in **Eqs 1**–**3** as the *PS* test, *SC* test, and *SP* test, respectively. The process of performing the *SP* test is shown graphically in **S2 Fig**. The overall process is the same for all three tests–they only differ in the statistic calculated.

These three statistical tests capture different aspects of how the distribution of characters observed along a lineage tree differs from random association between tree topology and trait values. The *PS* test determines the extent to which trait values are clustered together within the tree. A significantly low *PS* statistic (i.e. **δ** < 0, *p* < 0.05) indicates identical trait values are more closely clustered together within the tree than expected from random association between tree topology and trait values. By contrast, a significantly high *PS* statistic indicates identical trait values are less clustered together than expected by chance. Variations of this test were previously developed in [23] and applied in [30] to study spread of influenza.

While the *PS* test only determines a general association between trait values and tree topology, the *SC* and *SP* tests are both aimed at determining whether a particular trait value is more ancestral to another in the tree. A significantly high *SC* statistic (**Eq 2**) from state *i* to state *j* indicates a greater number of switches from state *i* to state *j* than expected from random association between tree topology and trait values. The *SC* test was used by [24] in the context of virus phylogeography, and by [6] to decompose the phylogenetic relationships among B cell subtypes within HIV infection. The *SC* test, however, is expected to have a high false negative rate because trees with randomized tip states often have more state changes events in general. Because of this, *SC* test **δ** values tend to be negative even when there is no polarity in the ancestor/descendant relationship among type *i* and *j* (**Fig 1**).

To normalize for differences in the total number of state changes between observed and permuted trees, we introduce the *SP* test. A significantly high *SP* statistic (**Eq 3**) from state *i* to state *j* indicates a greater proportion of switches from state *i* to state *j* than expected from a random distribution of trait values at the tips. In contrast to the *SC* test, a significant association between the tree and trait may exist, but only associations in which one trait is more often ancestral to the other than expected will give rise to a significantly high *SP* statistic (**Fig 1C**). Further, the denominator of the *SP* statistic can be altered to test other hypotheses. For instance, to test whether a greater proportion of state changes to state *j* come immediately from state *i* than expected by chance, one can restrict the analysis to consider only state changes towards *j*.

### Accounting for uncertainty in tree topology

To account for uncertainty in tree topology, we bootstrap the multiple sequence alignment of each clone [31]. This is performed by randomly sampling multiple sequence alignment columns with replacement. Lineage tree topology and branch length estimation then proceeds as before. Test statistics are calculated for each bootstrap replicate tree, and then for a single permutation of the traits at that tree’s tips. Calculations for **δ** values proceed as before, but with each *o*_*ij*_ value indexed for each replicate. This procedure is very similar to that proposed in [32] and can in principle be extended to other sets of tree topologies, such the posterior distribution of tree topologies generated by MCMC sampling under Bayesian phylogenetic inference.

### From trees to repertoires

B cell repertoire datasets often consist of hundreds or thousands of B cell lineage trees. Often hypotheses do not concern individual lineages, but instead the behavior of the collection of B cell lineages as a group. To characterize multiple B cell lineages, the observed and permuted summary statistics are summed across all lineages of a repertoire for each bootstrap replicate. Additionally, traits may be permuted among trees, which may increase statistical power and detect nonrandom association among trait types within trees.

### Simulations

We tested the performance of the three proposed statistics using simulations based on B cell lineage trees estimated from an empirical dataset. Sequences were obtained from peripheral blood samples taken from one subject at ten time points, from eight days before to 28 days after influenza vaccination [33] (subject 420IV). Sequence preprocessing and clonal clustering are described in [4]. Sequences were down-sampled by 50%, and only clones with >10 unique sequences were retained. A total of 399 clones containing 11 to 370 (mean = 26.2) unique sequences remained. Tree topologies and branch lengths were estimated for each clone using *dnapars* v3.967 [26] via the R package *alakazam* v0.3.0 [34] and *dowser* v0.0.1. We simulated state changing down each tree using a Markov model parametrized by initial frequencies ***π*** for each state, relative rate parameters *r*_*ij*_ for each pair of possible states *i* and *j*, and *r*, the average rate of state changes per mutation per site. The mean value of this rate matrix was calculated as the sum of the diagonal elements weighted by their initial state frequencies. All values of the matrix were then divided by this mean and multiplied by *r*. This calibration was performed so that r*l state change events are expected to occur across a branch of length *l* mutations/site under the initial state frequencies. Because the frequency of each state can change along the tree (i.e. the Markov chain is not necessarily at equilibrium), *r* may not necessarily equal the average rate of state change events further down the tree. For each tree, the state at the germline node was randomly drawn based on each state’s *π* value. For each node after the germline, the rate matrix is multiplied by the node’s ancestral branch length and exponentiated to give the probability of each state at that node, given the state at its immediate ancestor node. The state was then randomly chosen based on these probabilities. This process begins at the germline node and continues down the tree until each tip node has a state. Because each tip corresponds to a sequence, this forms a dataset of sequences paired with simulated discrete characters. Internal node states are not included in the final simulated dataset used for analysis. Simulations were performed with two state models (*A* and *B*) that explored a large parameter space (*π*_*a*_ = 0.5, 1; *r*_*ab*_ = 1, 10; *r* = 10, 25, 50, 100, 1000), and four state models (*A*, *B*, *C*, *D*) that explored more complex patterns of state change at low overall rate (*r* = 10). Twenty simulation repetitions were performed for each parameter combination. Statistical tests were performed as described in **Methods**; however, to improve computational efficiency simulation analyses did not use bootstrapped multiple sequence alignments, and instead performed 100 permutations on a fixed maximum parsimony tree for each clone. Only clones with more than one state type were analyzed.

To benchmark the performance of the *SP* test under more extreme conditions, we performed similar simulations along large phylogenies with asymmetrical topologies. In these simulations, perfect ladder topologies were used, with each internal node bifurcating to at least one tip and at most one internal node. All branch lengths were set to a length of 0.001 mutations/site. Simulations using these tree topologies used two states and otherwise continued as detailed above.

### Controlling the false positive rate using down-sampling

Initial analyses using simulations along large ladder phylogenies demonstrated that the *SP* test had a high false positive rate when the overall rate of switching was extremely low (**S6 Fig**). These problematic cases are identifiable as they result in trees with a high ratio of tips to predicted state changes (**S7 Fig**). We adjust for these cases by using a simple down-sampling algorithm. The goal of the down-sampling algorithm is to randomly draw a subset of the original data whose resulting phylogenetic tree cannot have a tip-to-state change ratio greater than a pre-specified value *k*. A tree containing *m* state types at its tips must have at least *m* -1 state changes along its edges. If a tree contains *m* state types and k*(*m*-1) tips, then the ratio of tips to state changes (which must be ≥ *m*-1) along its branches can be no larger than *k*. For the desired tip-to-state change ratio of *k*, the algorithm proceeds by randomly sampling one tip of each state type (*m*) from a tree, and then randomly sampling k*(*m*-1)–*m* remaining sequences without replacement. This produces a tree with *m* states, k*(*m*-1) tips, and a maximum tip-to-state change ratio of *k*. Tree building and summary statistic calculation then proceed as above. This down-sampling is repeated each repetition. For computational expediency, this process can be repeated on tree topologies that have been already estimated. Tips are simply sampled from the tree with the remaining topology and branch lengths intact. This down-sampling algorithm was implemented in *dowser* v0.1.0, and all analyses presented that use this algorithm use *dowser* v0.1.0, R v4.0.3 [35], and *ape* v5.5–3 (www.github.com/emmanuelparadis/ape, commit 4fd8482). All other analyses used *dowser* v0.0.1, R v3.6.1, and *ape* v0.5.3.

### Empirical datasets

We demonstrated the utility of the proposed discrete trait framework by analyzing two empirical datasets. The first was aimed at understanding B cell differentiation during HIV infection, and consisted of BCR mRNA sequences taken from sorted populations of unswitched memory B cell (MBC), CD19^hi^ MBC, CD19^lo^ MBC, and germinal center B cells (GCBC) from three HIV viremic subjects (subject 1–3; [6]). Each dataset was subsampled to a maximum of 50,000 total sequences, and only clones with more than 10 sequences were retained. Unique sequences associated with more than one cell type were kept distinct. This resulted in 138, 203, and 166 multi-cell type clones with a mean of 49.9, 39.4, and 30.3 unique sequences per clone, for subjects 1–3 respectively. State changes across all lineages for each subject were calculated over 100 bootstrap replicates. To control the false positive rate of the *SP* test, all trees were down-sampled to a maximum tip-to-state change ratio of 20.

The second dataset was aimed at understanding isotype switching patterns in human children, and consists of BCR mRNA sequences obtained from peripheral blood samples taken from a human subject each year from age 1 to 3 years old [36]. Preprocessing, including grouping of sequences into clonal clusters, is detailed in **S2 Appendix**. Only clones with at least 4 unique sequences and more than one isotype were retained. Identical sequences associated with more than one isotype were kept distinct so each sequence was associated with one isotype. This resulted in 792 multi-istoype clones with a mean of 9.1 unique sequences each. State changes across all lineages were calculated over 100 bootstrap replicates. To control the false positive rate of the *SP* test, all trees were down-sampled to a maximum tip-to-state change ratio of 20.

## Results

We outline three parsimony-based summary statistics to characterize the distribution of trait values along B cell lineage trees (**Fig 1**). The significance of these statistics is tested by comparing observed values within the set of trees that comprise a repertoire to those obtained from permuting trait values at the trees’ tips. The mean difference between each statistic calculated from the observed trees and permuted trees is referred to as **δ**. The process of calculating **δ** and *p* values is shown graphically for the *SP* test in **S2 Fig**. The first statistic, the parsimony score (*PS*), is the total number of trait value state changes that occurred along a lineage tree. A *PS* test with **δ** < 0 and *p* < 0.05 (*i*.*e*. a significantly low *PS* statistic) indicates the trait values cluster together in the observed trees more often than expected by chance (**Fig 1**). We propose two other statistics aimed at determining whether one state is more frequently the immediate ancestor to another state than expected by chance. The switch count (*SC*) from state *i* to *j* is the number of state changes that occurred from *i* to *j* [24], while the switch proportion (*SP*) from state *i* to *j* is the proportion of state changes that occurred from *i* to *j*. An *SC* or *SP* test from *i* to *j* with **δ** > 0 and *p* < 0.05 indicates trait value *i* was more frequently the immediate ancestor to state *j* than expected by chance. We expect the *SP* test to be more sensitive to this relationship than the *SC* test because it accounts for the higher number of state changes expected in randomized trees (**Fig 1**). Similarly, an *SP* test from *i* to *j* with **δ** < 0 and *p* < 0.05 indicates trait value *i* was less ancestral to state *j* than expected. All three of these tests may be used to characterize individual lineages or entire B cell repertoires; in this paper we will focus exclusively on repertoires.

### Differentiating state change patterns with two states

We used simulations to assess the performance of our proposed tests. We model B cell migration and differentiation using a Markov model with two states, *A* and *B*, and empirically-derived lineage tree topologies (**Methods**). Briefly, the pattern of state changes along a tree was determined by the probability that the state at the root was *A* (*π*_*a*_ = 0.5, 1; *π*_*b*_ *= 1 –π*_*a*_), the average rate of state change (*r* = 10, 25, 50, 100, 1000 changes/mutation/site), and the relative rate of change from *A* to *B* (*r*_*ab*_ = 1, 10; *r*_*ba*_ *= 1/r*_*ab*_). These parameters represent a range of slow, fast, biased, and unbiased state change patterns along a B cell lineage. Each simulation resulted in a dataset of BCR sequences, each associated with a single trait value (*A* or *B*) resulting from the simulation process. The goal of our simulation analysis is to determine if the summary statistics provide useful information about the mode and tempo of trait evolution.

We ran 20 simulation repetitions for each parameter combination, and tested the significance of each of the proposed statistics to assess their statistical power. Our simulations are designed to generate trees whose tip-states are more clustered together than if the tips states are randomly distributed across the tree tips. Consistent with this expectation, 320/320 simulation repetitions in which *r* < 1000 (*i*.*e*. overall rate of state change < 1000 changes/mutation/site) showed a significantly low *PS* statistic regardless of other parameters (**δ** < 0; *p* < 0.05; **S3 Fig**). This confirms the *PS* test’s usability for detecting nonrandom association between tree topology and trait values. However, at *r* = 1000, only 3/80 repetitions showed a significantly low *PS* statistic (**S3 Fig**), indicating this relationship is difficult to detect at high rates of state change.

We used the same simulations to test whether the *SC* statistic was capable of detecting the direction of state changes in B cell repertoires. A total of 300 simulation repetitions were performed using parameters expected to give biased (directed) state changes; namely, with lineages always beginning in *A* (*π*_*a*_ = 1) and/or highly biased rates of state change from *A* to *B* (*r*_*ab*_ = 10). Surprisingly, only 3/300 of these simulations showed a significantly high *SC* from *A* to *B* (**δ** > 0; *p* < 0.05; **S4 Fig**). By contrast, 186/300 showed a significantly low *SC* from *A* to *B* (**δ** < 0; *p* < 0.05). This indicates that significantly high *SC* statistics are highly conservative, while significantly low *SC* statistics are primarily driven by overall phylogenetic association with a trait. This issue is likely exacerbated as dataset size grows, hence the *SC* test is likely still useful for single lineages [24,25] or for detecting very strong trends in large datasets [6]. However, given these results the *SC* test does not appear appropriate as a general solution for detecting biased migration and differentiation in B cell repertoire datasets.

**Fig 2:**
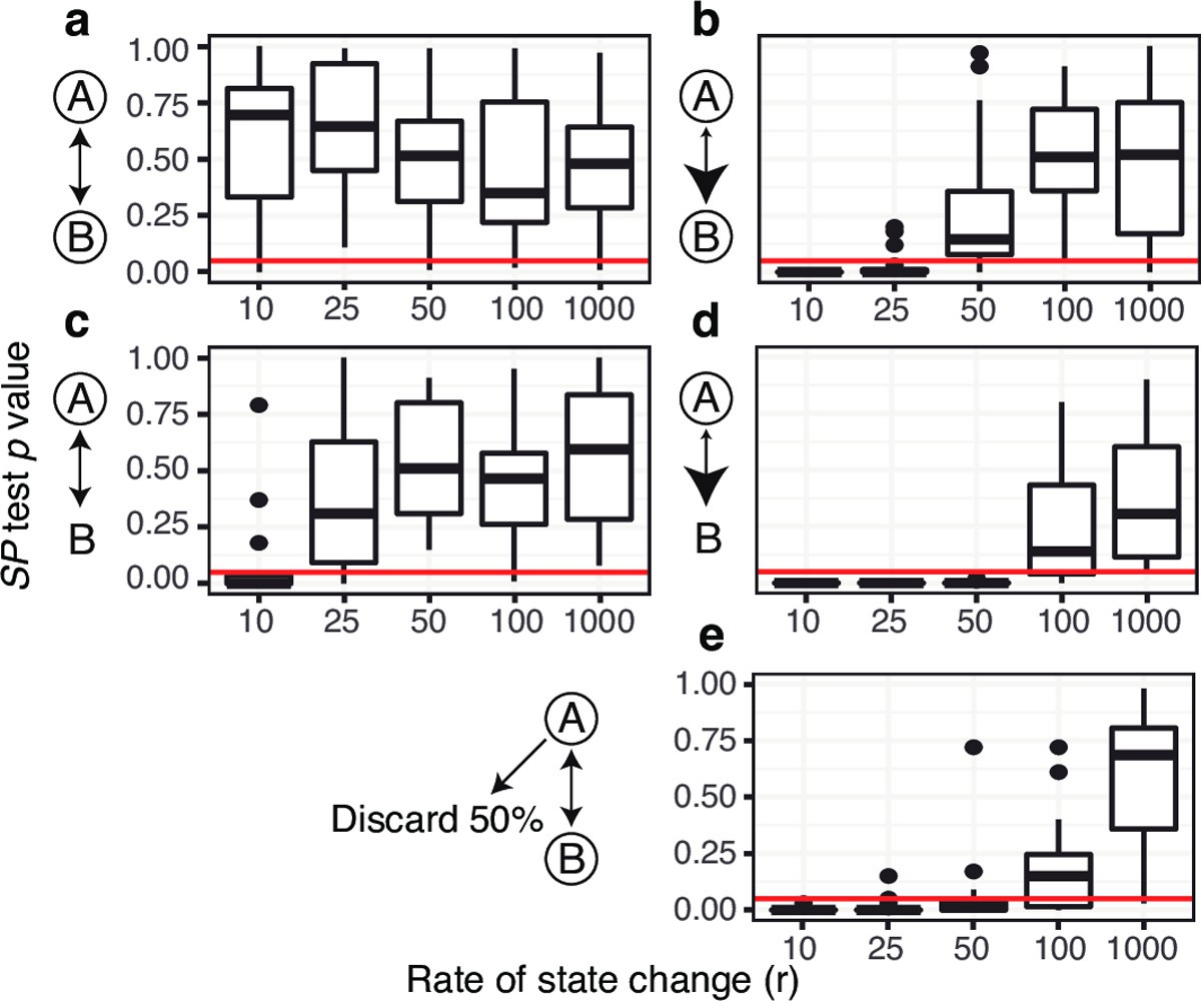
Distribution of *SP* test *p* values from *A* to *B* from two state simulation analyses in which state change between state *A* and *B* was determined by the probability of starting in *A* (*π*_*a*_), relative rate of migrating from *A* to *B* (*r*_*ab*_), and the average rate of state change (*r*). To the left of each plot, possible starting states are circled, relative rates are shown by arrowhead size. **(a)** *π*_*a*_ = 0.5, *r*_*ab*_ = 1, fully unbiased state change, shows roughly uniform distribution of *p* values at all tested rates. **(b)** *π*_*a*_ = 0.5, *r*_*ab*_ = 10 shows low *p* values at rates < 50. **(c)** *π*_*a*_ = 1, *r*_*ab*_ = 1 shows low *p* values at low rates (10) but not at higher rates. **(d)** *π*_*a*_ = 1, and *r*_*ab*_ = 10 shows low *p* values at rates < 100. **(e)** *π*_*A*_ = 0.5, *r*_*ab*_ = 1 shows low *p* values at rate < 50 if 50% of *A* sequences are discarded. Compared to **(a)**, this shows that *p* values are sensitive to biased sampling of sequences. Red lines show the cutoff of *p* value = 0.05.

We next tested whether biased state change patterns were detected by the *SP* test. To test this method’s false positive error rate, we first investigated simulations with totally unbiased state changes; namely, in which lineage trees were equally likely to begin at state *A* or *B* (*π*_*a*_ = 0.5) and had equal rates of state changes between *A* and *B* (*r*_*ab*_ = *r*_*ba*_ = 1). *SP* tests from *A* to *B* on these datasets resulted in a roughly uniform distribution of *p* values across all tested migration rates (**δ** > 0, *p* < 0.05 in 5/100; **Fig 2A**). This indicates that completely unbiased state changing is consistent with the null hypothesis of this test. Simulations in which lineages always had state *A* at the root (*π*_*a*_ = 1) and/or the relative rate of state change was higher from *A* to *B* (*r*_*ab*_ = 10) were expected to give high *SP* statistics. At low overall rates of state change (*r* = 10), 55/60 of these simulations had significantly high *SP* statistics from *A* to *B* (**δ** > 0; *p* < 0.05; **Fig 2B–2D**). As the rate of state change increased (*r* = 25, 50, 100, or 1000), this relationship diminished as the distribution of trait values became less distinguishable from random association (**Fig 2B–2D)**. These results indicate that, under this two state Markov model framework, a significantly high *SP* statistic is associated with biased origination, biased rate of state change, or both depending on the overall rate.

Finally, we used these simulated datasets to test whether the *SP* test is affected by biased data sampling, as this potential bias is important for some other phylogeographic methods of trait evolution e.g. [37]. We tested this by randomly discarding half of the sequences associated with *A* in simulations with totally unbiased state change (*π*_*a*_ *=* 0.5, *r*_*ab*_ = 1). Though *SP* tests from *A* to *B* on these datasets gave a uniform *p* value distribution when all sequences were included (**Fig 2A**), *SP* statistics from *A* to *B* became significantly high when half of *A* sequences were discarded (**Fig 2E**). This indicates that severely biased sampling may give a similar signature as biased origination or state change for the *SP* test (**Fig 2B–2D**). Biased sampling may be caused by a variety of experimental factors, and applications of these statistics to empirical datasets will need to carefully consider possible effects of biased data collection for each trait type.

### Differentiating complex relationships among trait values

**Fig 3:**
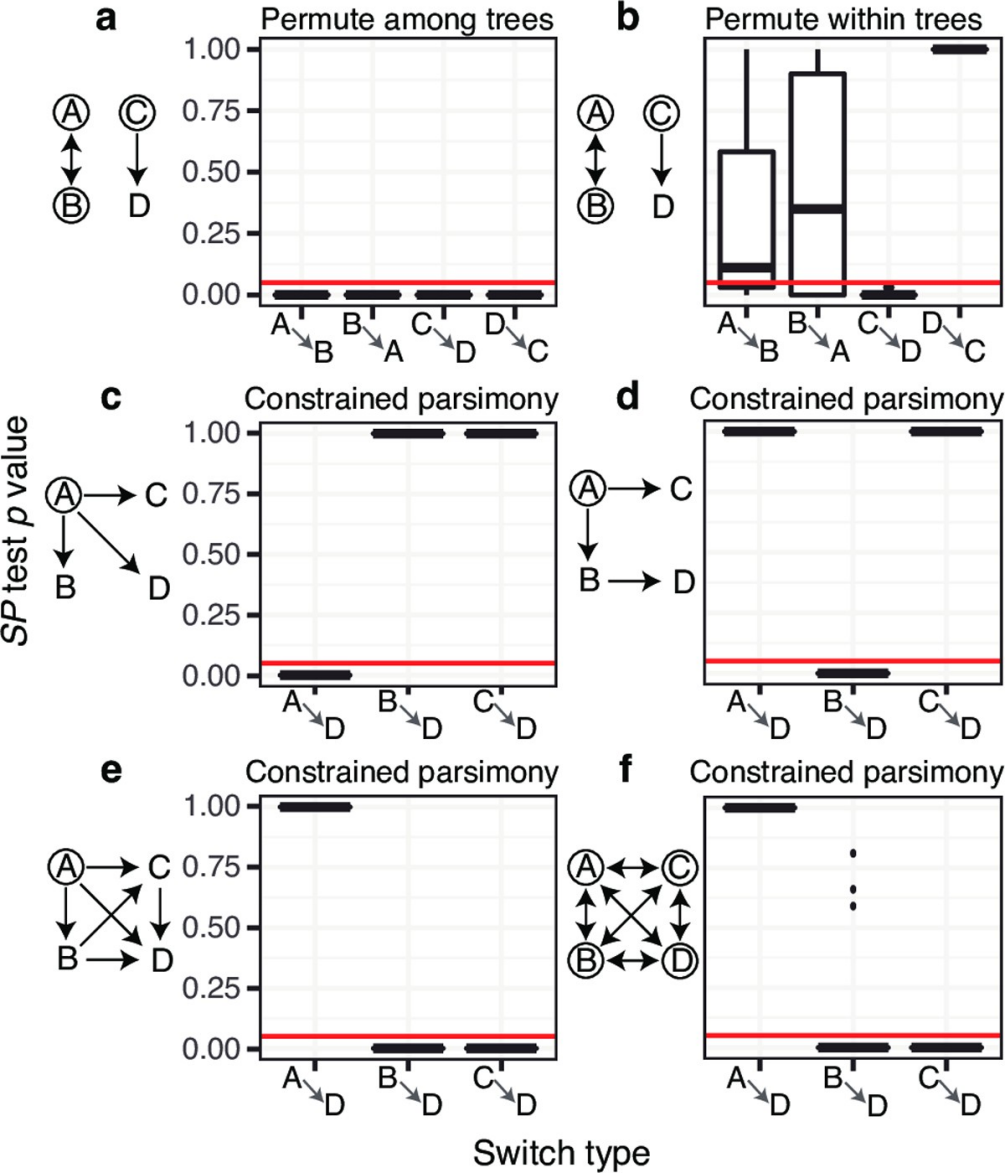
Distribution of *SP* test *p* values from four state simulation analyses under multiple modes of evolution diagrammed to the left of each plot. Twenty repetitions were performed in each scenario. In simulations, possible starting states are circled and possible state changes are shown with arrows. All allowed state changes occurred at the same relative rate and the total rate of state change (*r*) was 10 changes/mutation/site (see **Fig 2**). **(a)** Permuting trait values among trees reveals low *p* values for all state changes between *A* and *B*, and between *C* and *D*. **(b)** Permuting within each tree reveals low *p* values from *C* to *D*, but not between *A* and *B*. Both **a** and **b** imposed no constraints on the types of state changes allowed in the maximum parsimony algorithm. **(c)** Direct switching simulations result in low *p* values from *A* to *D*, but not from other states to *D*. **(d)** Sequential switching simulations result in low *p* values from *B* to *D* but not from other states to *D*. **(e)** Irreversible switching simulations result in low *p* values from *B* and *C* to *D*, but not from *A*. **(f)** Unconstrained switching simulations also result in low *p* values from *B* and *C* to *D*, but not from *A*. The strange results of **e** and **f** are likely artefacts of the constrained parsimony algorithm, which forbids reverse alphabetical state changes (e.g. *D* to *C*), used to count state changes in simulations **c-d**.

All the tests detailed above are extendable to data with more than two states; however, due to its superior performance in two state simulations, we will focus in the rest of this study on the *SP* test. The permutation step of the *SP* test usually permutes trait values within each tree separately (**Methods**). However, when more than two states are present it may be advantageous to randomize trait value assignments among trees rather than just within each tree. This changes the null hypothesis, which is now that the proportion of state changes observed is the same as that expected if trait values are randomly distributed among all trees. Deviations from this null hypothesis may be due not only to biased ancestor/descendant relationships within individual trees, but also co-occurrence of trait values within different trees. To demonstrate the difference between these two mechanisms, we performed simulations with four trait values: *A*, *B*, *C*, and *D*. To test the difference between simple association and biased ancestry, these simulations used unbiased state change between *A* and *B*, and unidirectional state change from *C* to *D*. Trees began with states *A*, *B*, or *C* in equal probability; state changes were allowed in both directions from *A* to *B* and unidirectionally from *C* to *D*. For each repetition, the rate of state change *r* = 10, and relative rates were equal among allowed state changes. Performing the *SP* test on these simulations while permuting among trees showed significantly higher *SP* statistics in both directions between *A* and *B*, and between *C* and *D* than expected (20/20 for each; **Fig 3A**). This indicates the *SP* test when permuting among trees detected the association between these trait values but not the directionality of *C* to *D* state changes. In contrast, the *SP* test when permuting only within trees correctly yielded a significantly high *SP* statistic from *C* to *D* in 20/20 simulations; further, no simulation yielded a significantly high *SP* statistic from *D* to *C*, indicating a low false positive rate (**Fig 3B**). No simulation using either permutation method showed a significantly high *SP* statistic between unassociated trait values (e.g. *A* and *C*), indicating a low false positive rate. These results indicate that permuting trait values within trees is a more effective means of detecting biased ancestor/descendant relationships, while permuting between trees is more appropriate for detecting associations among traits.

### Differentiating constrained modes of state change

In some instances, there are known constraints to the direction that state changes can occur, such as in isotype switching. Isotype-determining constant regions in humans are ordered as IgM/IgD, IgG3, IgG1, IgA1, IgG2, IgG4, IgE, IgA2. Human B cells begin with IgM/IgD, and because the mechanism of class switching is irreversible, these events can only occur sequentially in the order specified. For instance, IgA1 can switch to IgG4, but not to IgM or IgG1. This constraint may be naturally incorporated into the Sankoff parsimony algorithm [29] by making impossible isotype switches have an arbitrarily high weight. A frequent focus of isotype switching analysis is whether a particular isotype (e.g. IgE) arises from direct switching from IgM or from sequential switching from an intermediate isotype [9,10]. These types of hypotheses could be investigated using the *SP* test.

To determine if the *SP* test can usefully distinguish between types of constrained relationships among trait values, we simulated datasets to represent possible isotype switching patterns. As above, datasets contained four trait values: *A*, *B*, *C*, and *D* under different modes of evolution. Because questions often focus on the origin of a particular isotype [10] we only counted state changes leading to *D* when calculating *SP* statistics. Further, because state changes can only occur in a particular direction, we permute trait values among trees in these tests to increase power. While we previously showed that permuting among trees confuses biased association with biased ancestry (**Fig 3A**), switching between these states can only occur in one direction. Because of this, association between two states implies a direction of switching and among tree permutation is justifiable. We first simulate direct switching in which trees always had state *A* at the root and only state changes from *A* to the other states were allowed (**Fig 3C**). We expected these simulations to show a significantly high *SP* statistic only from *A* to *D*. Confirming this expectation, all 20 of these simulations had a high *SP* statistic from *A* to *D* (**δ** > 0, *p* < 0.05; **Fig 3C**). We next simulated sequential switching, where arriving at state *D* requires transitioning through *B*. All trees began in *A* and state changes were allowed from *A* to *B* and *C*, but *D* arose only from *B*. We expected these simulations to show a significantly high *SP* statistic only from *B* to *D*. All 20 of these simulations showed a significantly high *SP* statistic from *B* to *D* (**δ** > 0, *p* < 0.05; **Fig 3D**). These results demonstrate that the *SP* test using constrained parsimony can discriminate between simple hypotheses of isotype switch patterns, such as direct versus sequential switching.

We next investigated whether the *SP* test can distinguish between more complex types of constrained switching. We simulated irreversible isotype switching in which trees begin with state *A*, and only state changes moving alphabetically (*A* to *D*) were allowed. Naively, we may expect these simulations should show similar *SP* test results from *A*, *B*, and *C* to *D*. However, all 20 of these simulation repetitions showed a significantly high *SP* statistic to *D* from *B* and *C*, but not from *A* (**Fig 3E**). As a control, we simulated unconstrained switching in which trees begin randomly at any state and may change between all states. Using a constrained parsimony model, these simulations showed the same significantly high *SP* to *D* from *B* and *C*, but not from *A* (**Fig 3F**), indicating that this pattern is possibly an artifact of the constrained parsimony model. These results demonstrate that, while the *SP* test outlined here can distinguish between simple types of constrained state change, its relationship to more complex modes of constrained state change such as irreversible evolution are difficult to predict, and should be interpreted cautiously.

### Controlling false positives in the case of slow rates of state change

Simulations based on empirical data have shown that the *SP* test can reliably detect biased state change along phylogenetic trees in many cases (**Figs 2** and **3**). However, because the null hypothesis of the *SP* test is that trait values are randomly distributed along tree tips, the *SP* test may have a high false positive rate in situations where the total number of predicted state changes along the tree is significantly different than expected from the null hypothesis. One such case is that of slow state change along a large tree. We confirmed this using 50 repetitions of two-state simulations using unbiased (*π*_*a*_ = 0.5, *r*_*ab*_ = 1) switching along a single ladder phylogeny of 1000 tips and a slow rate of state change *r* = 1 state change/mutation/site (**Methods, S6 Fig**). Though the switching process in these trees was unbiased, 42% of repetitions had a significantly high *SP* statistic from *A* to *B*, and 32% had a significantly high *SP* statistic from *B* to *A*, for a total false positive rate of 74%. This indicates that, without correction, the *SP* test is unreliable when the rate of state change is slow and the tree is large.

We investigated whether the issue of slow state change rates over large trees was a significant problem in empirical datasets by characterizing the ratio of tips-to-state transitions for each tree. Slow rates of state transition are expected to produce fewer state transitions along a tree. Simulations using ladder phylogenies confirm this prediction: simulations using a slow rate of state change (*r* = 1) had a mean tip-to-state change ratio of 326.8; however, when *r* = 10 this ratio dropped to a mean of 37.6, and when *r* = 100 this ratio was only 5.2 (**S7 Fig**). We next calculated the tip-to-state change ratio in three empirical datasets in lineages with at least 20 sequences. The first of these characterized cell type transitions in HIV infection, and had a mean tip-to-state change ratio of 33.4. The second empirical dataset focused on isotype switching in a food allergic patient, and had a mean tip-to-state change ratio of 11.1 (**S7 Fig**). Finally, we included a recently published dataset of B cells obtained from blood and thymus samples from myasthenia gravis patients [8], which had a mean tip-to-state change ratio of 35.4 (**S7 Fig**). These results show that rates of state change in empirical BCR datasets are characteristic of simulations in which *r* is between 10 and 100 state changes/mutation/site. The *SP* test performs well under these rates (**Fig 2**). Collectively, these results show that trees with rates of state change that are problematically slow for the *SP* test are identifiable in practice by calculating the ratio of tips to predicted state changes, and uncharacteristic of the BCR datasets analyzed in this study.

**Fig 4:**
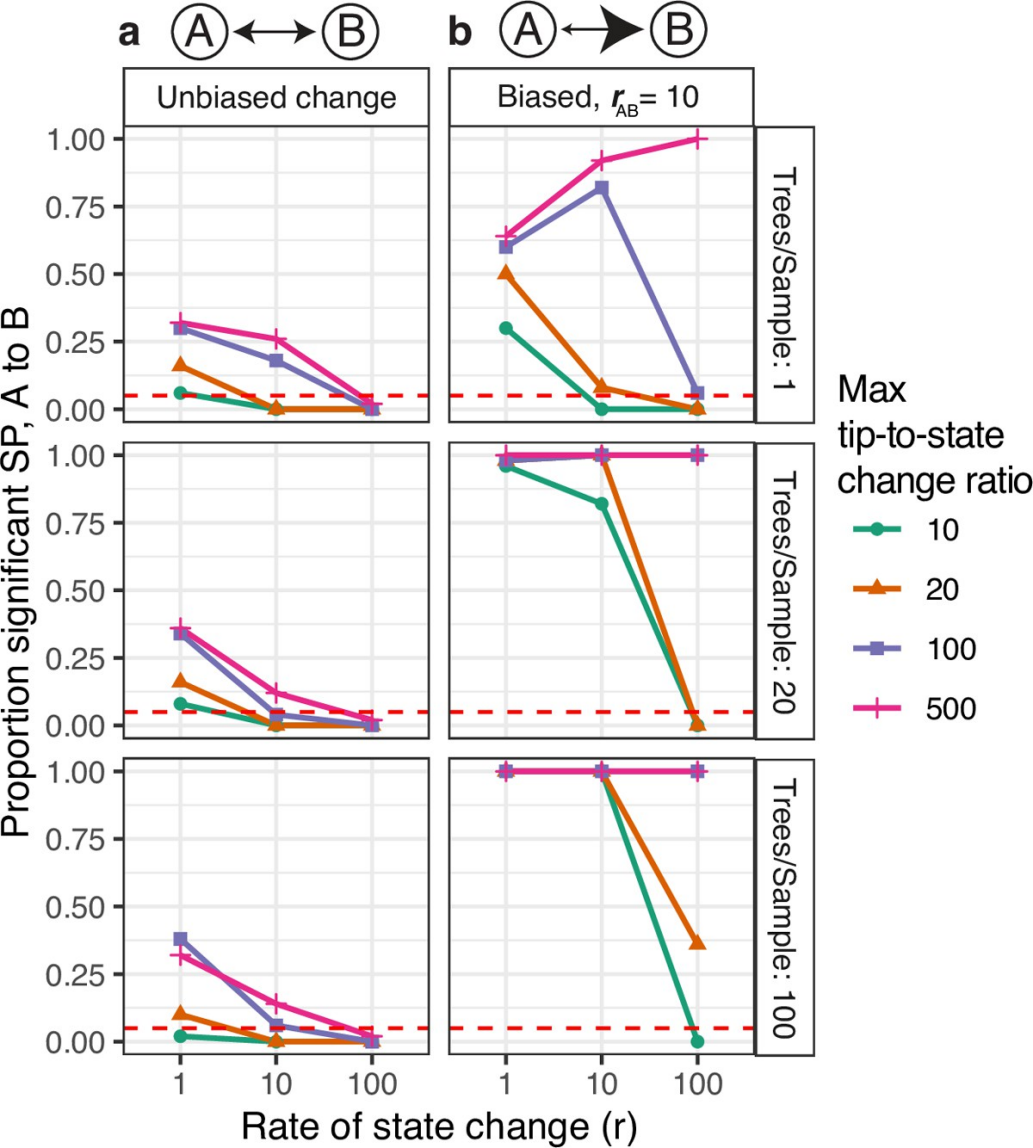
Controlling false positive rates using down-sampling. Y axis shows the proportion of 50 simulation replicates in which the *SP* test from *A* to *B* was significant. Simulations were performed on large ladder phylogenies (**Methods**) at varying rates, biases, and trees per repetition (representing lineages within a simulated repertoire). In each case, simulated lineages were down-sampled to the specified maximum tip-to-state change ratio. **(a)** Unbiased simulations, significant *SP* test values indicate false positives. These show that the *SP* test has a high rate of false positives when the rate of state change is low (i.e. 1 change/mutation/site) and the maximum tip-to-state change ratio is high (> 20). Down-sampling lineages to a maximum tip-to-state change ratio of 10–20, however, controls this false positive rate. **(b)** Simulations with rates biased from *A* to *B*, in which significant *SP* test values indicate true positives. These results show that down-sampling does not simply reduce power. However, power is lowered when the switching rate is high (>100) or the number of trees/repertoire is low (< 20).

To further address the issue of poor performance during slow rates of state change, we developed a simple down-sampling algorithm to control the false positive rate when the rate of state change (*r*) is low (**Methods**). This algorithm randomly down-samples each clonal lineage such that its ratio of tips to state changes is less than or equal to a pre-specified threshold. To assess whether this controlled the false positive rate, we first performed simulations in which the state change process was completely unbiased. In these simulations, the proportion of repetitions with a significantly high *SP* test statistic from *A* to *B* indicated the rate of false positives from *A* to *B*. As expected, the false positive rate was high when the ratio of tips to state changes was high. At a tip-to-state change ratio of 500, 32% of repetitions simulated with *r* = 1 had a significantly high *SP* test statistic from *A* to *B* in repertoires with 100 trees (**Fig 4A**). When these same lineages were down-sampled to a maximum ratio of 10, however, the false positive rate from *A* to *B* was only 2%, less than expected given the alpha value of 0.05. To test if down-sampling to a low tip-to-state change ratio led to a significant reduction in power, we repeated these analyses with simulations in which the relative rate of switching from *A* to *B* (*r*_*ab*_) was 10. In these simulations, repetitions with significantly high *SP* statistics from *A* to *B* represent true positives. We found that at all levels of down-sampling, simulations with biased (*r*_*ab*_ = 10) and slow rates of state change (*r* = 1 and 10) had true positive rates ranging from 82% to 100% in repetitions containing at least 20 lineages (**Fig 4B**). This demonstrated that down-sampling did not significantly lower power in these cases. Down-sampling to a ratio of 10–20 did have low power when performing analyses in faster rates of state change *r* = 100 (mean true positive = 6.0%) or when there was only one tree within a simulated repertoire (mean true positive = 14.7%). Down-sampling also did not increase the false positive rate of *SP* test from *B* to *A* in the simulations tested (**S8 Fig**). Repeating previous simulations (**Fig 2**) with down-sampling to a tip-to-state change ratio of 20 showed consistent results, with a reduction in power at higher rates of state change (**S9 Fig**). Collectively, these results suggest that ensuring all trees analyzed have a maximum tip-to-state change ratio of 10–20 adequately controls the false positive rate in the case of slowly changing discrete states without severely limiting power. This could be achieved by down-sampling lineages with a higher ratio of tips to state changes.

### Differentiation of B cell subtypes during HIV infection

Over the course of the immune response, B cells differentiate into multiple cellular subsets with distinct properties. Recent studies have focused on the role of T-bet, a transcription factor usually associated with differentiation of T cells, in shaping B cell responses during infection. For example, [6] used data from three HIV+ patients to demonstrate that CD19^hi^ memory B cells (CD19^hi^ MBCs, a surrogate for T-bet+ B cells) represented earlier states in the affinity maturation process than germinal center B cells (GCBCs), and to define the relationships among other B cell subtypes including CD19^lo^ MBCs and un-switched MBCs. More specifically, [6] used the *SC* test with trait values permuted among trees. However, the simulation analyses performed here demonstrated the *SC* test is highly conservative, and that permuting among trees may only detect unstructured association among trait values (**Fig 3**). It is therefore not clear whether the relationship from CD19^hi^ MBCs to GCBCs observed in [6] was driven by biased ancestor/descendant relationships among these cell types within trees. Our results above suggests that the *SP* test using within tree permutation would be a more appropriate test of this relationship.

**Fig 5:**
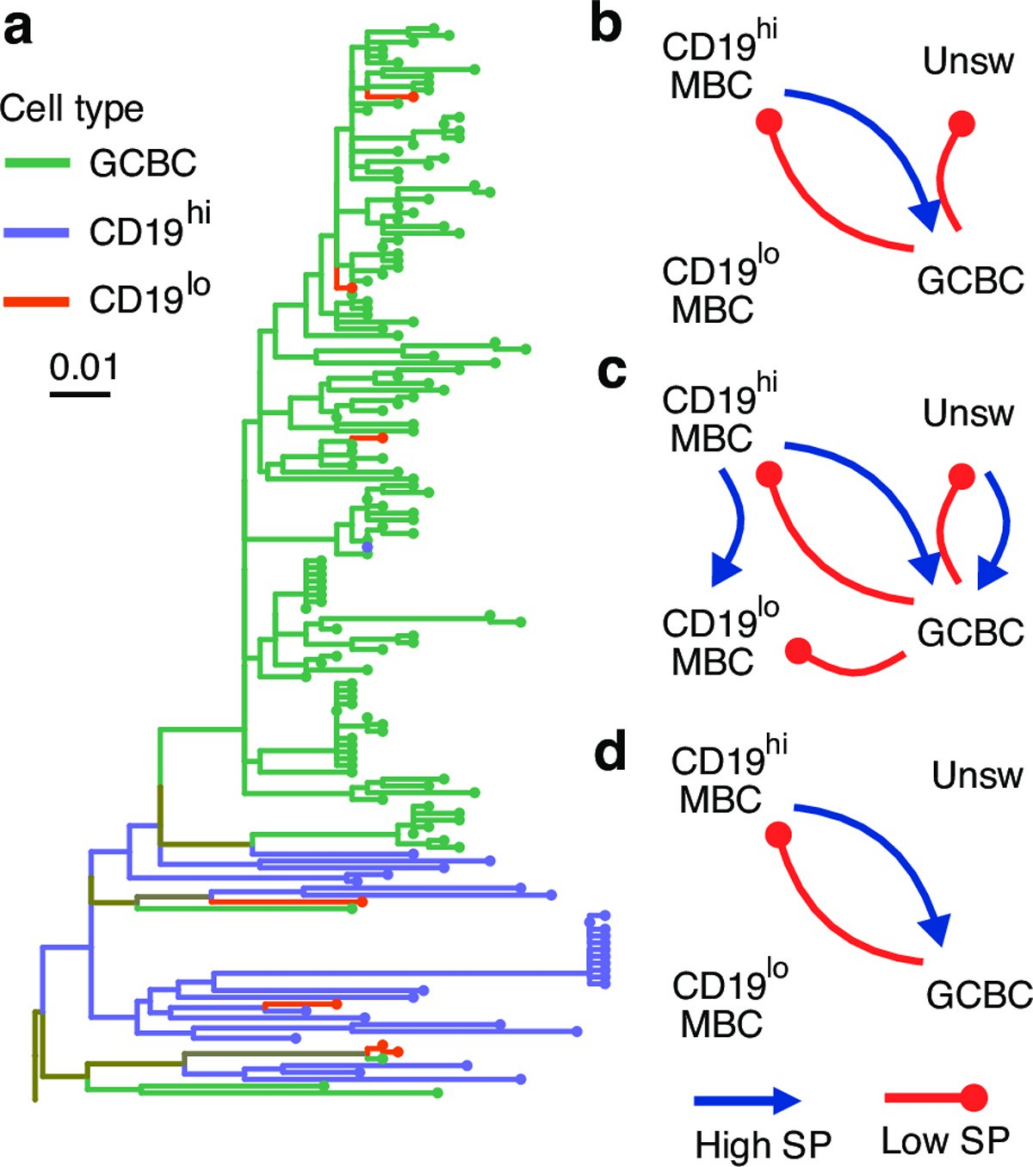
Analysis of B cell subtypes in three HIV+ subjects. **(a)** Example tree visualized using *ggtree* [38,39] showing observed relationship between CD19^hi^ MBCs and GCBCs. **(b-d)** Direction of significant *SP* test **δ** values for subjects 1 **(b)**, 2 **(c)**, and 3 **(d)**. Arrows within each diagram show the direction of significantly high (blue) or significantly low (red) *SP* statistics between CD19^hi^ MBCs, CD19^lo^ MBCs, unswitched MBCs (Unsw), and GCBCs in each subject.

We characterized the relationships among B cell subsets with a two-tailed *SP* test using within tree permutation for each of the three subjects. These analyses showed a significantly high *SP* statistic from CD19^hi^ MBCs to GCBCs in all three subjects, and to CD19^lo^ MBCs in one subject (**δ** > 0, *p* < 0.025; **Figs 5B–5D and** S10). These analyses confirm the conclusions in [6] that CD19^hi^ MBCs are significantly closer, cladistically, to the predicted germline sequence than GCBC sequences than expected by chance. Naively, one may interpret this as evidence that GCBCs derive from CD19^hi^ MBCs. However, because GCBCs are expected to have far higher mutation rates than MBCs, the observed patterns are also consistent with early production of CD19^hi^ MBC from GCBCs, followed by a near cessation of mutations in CD19^hi^ MBCs. This is consistent with the conclusions of [6] that CD19^hi^ MBCs represent earlier stages in the GC reaction, rather than the direction of differentiation. Overall, these *SP* tests confirm that the previously observed relationships from CD19^hi^ MBCs and CD19^lo^ MBCs to GCBCs are driven by biased ancestor/descendant relationships within trees rather than simply association in the same trees, as may have been the case from the previously used *SC* tests with among tree permutations [6].

### Sequential isotype switching to IgE and IgG4

Antibody isotypes are a major determinant of function. Of particular interest is characterizing whether IgE antibodies, the primary antibody isotype associated with allergic response, arise directly from IgM switching, or through sequential switching from another downstream isotype [9,10]. Previous studies have shown evidence that IgE in mice and human adults arises from sequential switching primarily from IgG [9,10], though a recent study in 27 humans in the first three years of life found evidence of a greater association between IgA1 and IgE in children with food allergy and eczema [36]. Specifically, [36] showed a higher number of shared clones between IgE and IgA1 than between IgE and other isotypes in these subjects. A phylogenetic test of this relationship would confirm that IgE and IgA1 sequences show a direct ancestor-descendant relationship within these B cell trees rather than just being part of the same clone.

**Fig 6:**
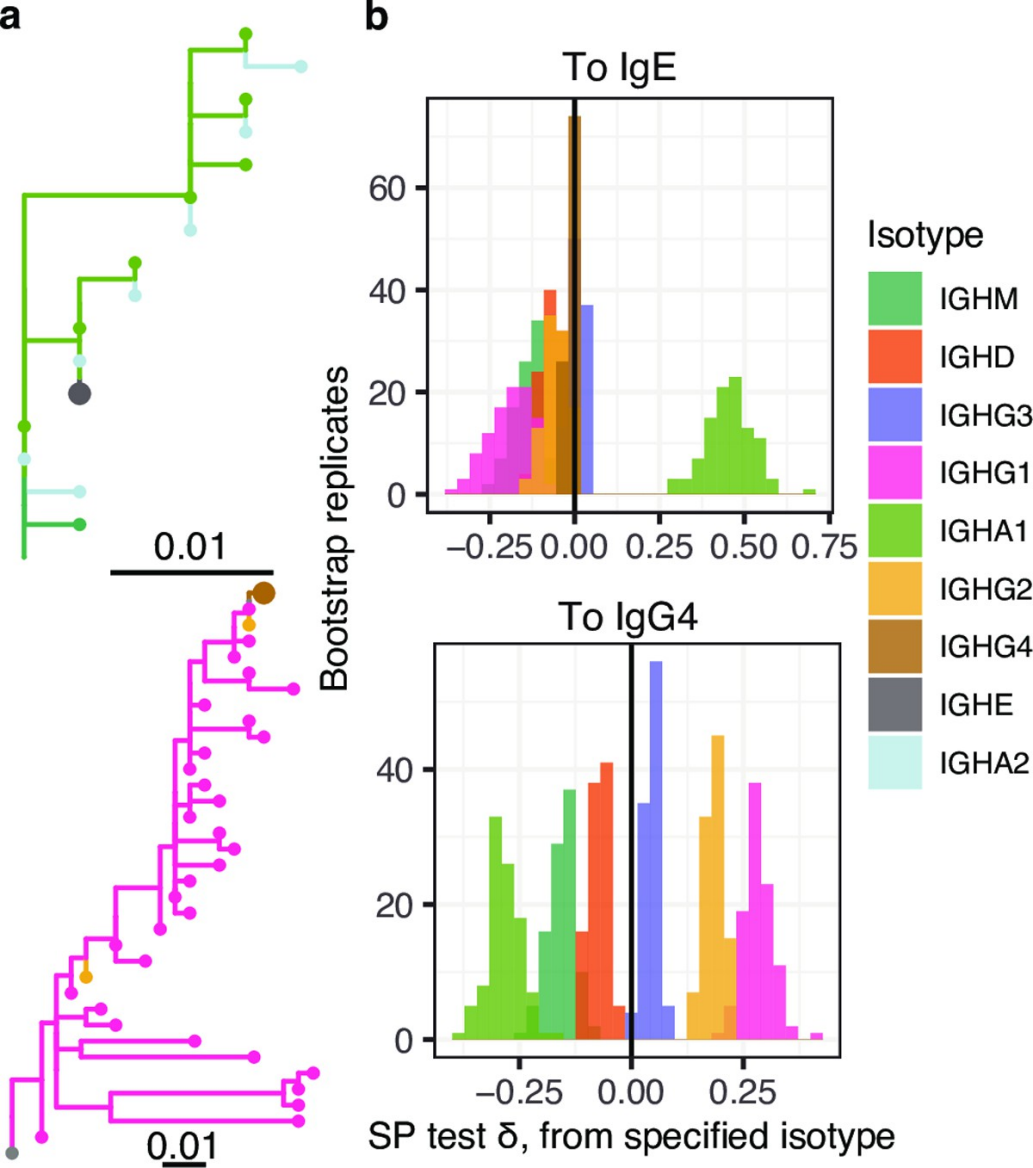
Analysis of antibody isotypes from a single subject. **(a)** Example trees visualized using *ggtree* [38,39] showing observed relationships between cells expressing BCRs with IgA1 and IgE, as well as IgG1 and IgG4 isotypes. IgE and IgG4 are indicated on each tree using larger tip circles. **(b)** Distribution of *SP* test δ values to IgE from each of the other isotypes (different colors), and distribution of *SP* test δ values to IgG4 from each of the other isotypes (different colors). State changes in **b** were calculated using constrained parsimony which forbids state changes that violate the geometry of the Ig heavy chain locus, and *SP* tests were performed using permutation among trees.

We applied our discrete trait framework to determine the origins of IgE in a single subject (id = 2442) from [36]. This subject was selected due to reported history eczema, food allergy, and B cell clones containing IgE and other isotypes [36]. Using an *SP* test in which only state changes leading to IgE were considered and trait values were permuted among trees, we found a significantly high *SP* statistic from IgA1 to IgE (**Fig 6B**). No other isotype showed a significantly high *SP* statistic to IgE. These results favor IgE arising from sequential switching through IgA1 over direct switching from IgM in this subject. Performing a similar test using only state changes leading to IgG4 revealed a significantly high *SP* statistic from IgG1, IgG2, and IgG3 to IgG4 (**Fig 6B**). This pattern is similar to irreversible switching within the IgG family (**Fig 3E**). As shown in simulation analyses, this test is not suited to infer relative rates of switching from different isotypes if all kinds of switches are considered. However, these results are most consistent with origin of IgG4 through sequential switching with other IgG isotypes rather than direct switching from IgM or sequential switching from IgA1. Overall, these results are consistent with the conclusions of [36] that IgE arose preferentially through sequential switching from IgA1 in this subject. Our results further suggest IgG4 arose preferentially through sequential switching from other IgG subtypes in this subject.

## Discussion

Phylogenetic techniques have the potential to reveal important information about B cell migration, cellular differentiation, and isotype switching. While great strides have been made using phylogenetic models to study evolution and trait change generally, there are significant challenges to translating these approaches to B cells. As a step in this direction, hypotheses about the ancestor/descendant relationships of B cell trait values may be usefully investigated using heuristic approaches that are robust to uncertainties in branch length estimation. Here, we outline three maximum parsimony-based summary statistics (two of which were used in prior studies) to characterize the distribution of trait values along phylogenetic trees. Significance of all of these statistics is calculated using a permutation test. We demonstrate the efficacy of these tests using simulations, and show that the *SP* test is the most useful for characterizing ancestor/descendant relationships among trait values. We further demonstrate how these statistics can test hypotheses about empirical B cell datasets by characterizing the relationship between T-Bet+ memory B cells and germinal center B cells in three HIV+ patients, and the class switching origins of IgE and IgG4 in a human subject during the first three years of life.

Simulations demonstrate that the *SP* test was uniquely able to determine the direction of biased origination and state change among the approaches investigated. In simple simulations containing two states (*A* and *B*) a significantly high *SP* statistic from *A* to *B* was associated with origination in *A* and biased state change from *A*. This signal decreased as the overall rate of switching increased. In more complex scenarios, the *SP* test was able to differentiate between traits that were generated through biased state change in a particular direction versus traits that were simply associated with each other. The *SP* test was also able to distinguish between simple modes of constrained evolution such as direct and sequential switching. These results indicate the *SP* test may have broad utility in characterizing ancestor/descendant relationships among B cell discrete traits.

We next used two datasets to demonstrate that the *SP* test could be used to derive meaningful biological conclusions. In the first, we confirm that T-Bet+ memory B cells tend to be the predicted immediate ancestors of GC B cells within lineages trees obtained from three HIV+ subjects. Though this relationship may primarily be due to differences in mutation rate over time between memory and GC B cell subsets, this does confirm prior findings and demonstrates that the T-Bet+ memory B cell subset represents an earlier state in the affinity maturation process, possibly contributing to an impaired immune response to HIV [6]. We next characterized the isotype switching patterns of sequences obtained from a human over the first three years of life [36]. In this analysis, we found evidence of sequential switching from IgA1 to IgE, as well as evidence of sequential switching from IgG subtypes to IgG4. Sequential switching from IgA1 to IgE is consistent with [36] but not other analyses performed on data taken from adults, which favor sequential switching from IgG [10]. This possibly reflects differences in isotype switching patterns between adults and children. Overall these results demonstrate that the discrete trait analysis framework developed here can be used to test important hypotheses about B cell differentiation and class switching.

There are a number of limitations with these methods. First, tree topologies were estimated using maximum parsimony. While maximum parsimony is not a statistically consistent estimator of tree topology and is known to give inaccurate predictions over long branch lengths [40], it has been shown to be an accurate estimator of tree topology in certain B cell applications [27], and is widely used in B cell phylogenetic analysis [5,22]. In any case, the statistics presented here are not limited to tree topologies inferred through maximum parsimony, and parsimony was used primarily for computational expediency. The three statistics proposed here (**Eqs 1**–**3**) are also based on maximum parsimony, and may have similar inaccuracies over long branch lengths. Further, the statistical tests assume that the process of state change is independent of the tree shape, when the two may be coupled e.g. [41]. This assumption of independence is commonly made in discrete trait analysis e.g. [13] to enable computational tractability, and because the actual link between tree shape and state change is unknown or cannot be modeled. A significantly high *SP* statistic could be potentially caused by factors other than biased state change. For instance, because tree branch lengths represent genetic distance rather than time, it is possible that cell types with low mutation rates over time will spuriously appear ancestral to those with high mutation rates. As discussed previously, this effect likely underlies our analysis of B cell subtypes in HIV infection. Finally, it is possible that SHM is actually occurring at another, un-sampled site which is seeding the sites that were sampled (**S1 Fig**). Overall, it is important to carefully consider alternative explanations when trying to determine the biological basis for a significantly high *SP* statistic.

An important limitation of the *SP* test is that, like many other phylogeographic approaches e.g. [37], it is affected by biased data sampling. This may arise due to experimental factors that are difficult to control. For instance, under-sampling a trait value may cause a spurious, significantly high *SP* statistic from that trait value. Previous analyses of viral migration have dealt with potential sampling bias by performing tests across multiple down-sampling repetitions [14]. In practice, it can be difficult to know if B cells with certain trait values have been sampled proportionally to their relative population sizes. However, if a type of B cell is known to be under-sampled in a particular experiment, and is predicted to be the descendant of another B cell type, it can be argued that this relationship is unlikely to be due to biased sampling (**Fig 2E**). Alternatively, if multiple samples are tested it is possible that these samples will have a wide range of sequence proportions belonging to different traits. If these differences in sequence proportions are uncorrelated with *SP* test results, it could be argued that observed results are unlikely to be due to consistent under-sampling of B cells with a particular trait value.

Our simulation analyses revealed that the *SP* test is difficult to interpret when considering complex constrained models such as irreversible isotype switching (**Fig 3**). To recreate isotype switching, we performed four state simulations in which only state changes proceeding in the direction of state *A*, *B*, *C*, and *D* were allowed. Unexpectedly, these simulations tended to show a significantly low *SP* statistic from *A* to *D*, but a significantly high *SP* statistic for *B* and *C* to *D* (**Fig 3E**). This biased trend is likely driven by the fact, due to constraints in the direction of state change, randomized trees tend to have more switches from *A* than expected based on the relative frequency of *A*. This produces a significantly high *SP* for switches from *A* to *D*. An alternative may be to use the *SP* statistic (**Eq 3**) without comparing to a null distribution, which is equivalent to comparing the relative frequency of each type of switch observed. However, the observed switch frequency (*SP* statistic) is not proportional to the true relative rate of state change in general. For instance, in the two state Markov model simulations presented here (**Fig 2**), the *SP* statistic alone is both positively and negatively related to the true relative rate of state change, depending on other parameters **(S5 Fig**). Comparing *SP* statistics to those obtained from randomized trees (i.e. the *SP* test) usefully corrects this relationship in unconstrained models (**Fig 2**), but not always in constrained ones (**Fig 3C–3E**). Ultimately, isotype switching is a complex, constrained process, and our analyses suggest the relative rates of isotype switching inferred from B cell trees should be interpreted cautiously. We suspect a general method for accurately estimating these rates will require a model-based approach, such as a non-reversible Markov model.

Finally, it is important to note that the *SP* test can have a high false positive rate when the rate of state change is very slow relative to the rate of mutations. This is because the null hypothesis of the test is that trait values are randomly distributed along each tree’s tips. This is convenient to calculate, but makes the test sensitive to cases where state changes are produced in an unbiased manner but where the total number of state changes along the tree is significantly different than that expected from the null hypothesis. A slow rate of state change along a large tree has this effect, and the *SP* test performs poorly in this case (**Figs 4 and S6**). However, we show that i) trees with slow rates of state change are identifiable in practice by calculating the ratio of tips to predicted state changes (**S6 and S7 Figs**) ii) trees with problematically slow rates of state change are uncharacteristic of the real B cell lineage trees surveyed (**S7 Fig**), and iii) the false positive rate from this source can be controlled using a simple down-sampling algorithm (**Figs 4 and S8 and S9**). In practice, we recommend always down-sampling all trees to a tip-to-state change ratio ≤ 20. This feature is included in *dowser* v0.1.0.

Future methods to differentiate migration, differentiation, and isotype switching patterns in B cells might improve upon the approach developed here by explicitly modeling these processes along a phylogeny, incorporating branch length information, and better accounting for uncertainty in tree topology. The heuristic approach introduced here crucially does not use branch lengths to help predict internal node states of the tree. Ignoring this source of information likely lowers power, but is possibly advantageous because the relationship between mutation rate and time is not currently well understood, and likely varies by cell type. While the approach developed here uses phylogenetic bootstrap replicates to account for uncertainty in tree topology [31], this may also be done using a posterior distribution of topologies generated by MCMC sampling. This was recently done for naive sequence inference in individual B cell lineages [42]. Phylogenetic bootstrapping has less desirable statistical properties than posterior distributions, but is a widely used means of assessing reproducibility of tree topology and is more computationally tractable for large datasets. Overall, though there is potential for improvement, the approach introduced here effectively deals with important challenges such as incorporating information across trees, accounting for uncertainty in tree topology, and scaling efficiently when analyzing large datasets.

A phylogenetic discrete trait analysis framework fills an important gap in B cell sequence analysis. The proposed framework provides a principled, flexible, and scalable approach for characterizing migration, cellular differentiation, and isotype switching in a wide array of contexts. This differs from other phylogenetic tools we developed recently, which used model-based approaches for characterizing somatic hypermutation and clonal selection [4,28].

## Supporting information

S1 Appendix Maximum ambiguity resolution of polytomies. (PDF)

S2 Appendix Processing of empirical datasets. (PDF)

S1 Fig Possible mechanisms of tracking B cell migration through somatic hypermutation. **(a)** Direct migration from tissue *A*, followed by extrafollicular mutation in tissue *B*. **(b)** Migration from tissue *A*, through a germinal center where SHM accumulates, and then migration to tissue *B*. **(c)** Early germinal center exit to tissue *A*, late germinal center exit to tissue *B*. This is not actually migration between tissue *A* and *B*. **(d)** Inferred phylogenetic tree in all three cases showing tissue *A* as the likely ancestral state compared to tissue *B*.
(PDF)

S2 Fig Visual diagram of *SP* test calculation on a single lineage tree. (PDF)

S3 Fig Simulation analyses with *PS* test. Distribution of *PS* test *p* values for the hypothesis that **δ** < 0 from two state simulation analyses. See **Fig 2** for analysis of the same data with the *SP* test. In these simulations, change between state *A* and *B* was determined by the probability of starting in *A* (*π*_*a*_), relative rate of migrating from *A* to *B* (*r*_*ab*_), and the average rate of state change (*r*). To the left of each plot, possible starting states are circled, relative rates are shown by arrowhead size. **(a)** *π*_*a*_ = 0.5, *r*_*ab*_ = 1, fully unbiased state change. **(b)** *π*_*a*_ = 0.5, *r*_*ab*_ = 10. **(c)** *π*_*a*_ = 1, *r*_*ab*_ = 1. **(d)** *π*_*a*_ = 1, and *r*_*ab*_ = 10. **(e)** *π*_*A*_ = 0.5, *r*_*ab*_ = 1, 50% of *A* sequences are discarded. Red lines show the cutoff of *p* value = 0.05.
(PDF)

S4 Fig Simulation analyses with *SC* test. Distribution of *SC* test *p* values for the hypothesis that **δ** > 0 from two state simulation analyses. See **Fig 2** for analysis of the same data with the *SP* test. In these simulations, change between state *A* and *B* was determined by the probability of starting in *A* (*π*_*a*_), relative rate of migrating from *A* to *B* (*r*_*ab*_), and the average rate of state change (*r*). To the left of each plot, possible starting states are circled, relative rates are shown by arrowhead size. **(a)** *π*_*a*_ = 0.5, *r*_*ab*_ = 1, fully unbiased state change. **(b)** *π*_*a*_ = 0.5, *r*_*ab*_ = 10. **(c)** *π*_*a*_ = 1, *r*_*ab*_ = 1. **(d)** *π*_*a*_ = 1, and *r*_*ab*_ = 10. **(e)** *π*_*A*_ = 0.5, *r*_*ab*_ = 1, 50% of *A* sequences are discarded. Red lines show the cutoff of *p* value = 0.05.
(PDF)

S5 Fig *SP* statistics in two state simulation analyses. Distribution of raw *SP* statistics from *A* to *B* in two state simulations. The red line is at 0.5, showing equal switch frequency. See **Fig 2** for analysis of the same data with the full *SP* test. In these simulations, change between state *A* and *B* was determined by the probability of starting in *A* (*π*_*a*_), relative rate of migrating from *A* to *B* (*r*_*ab*_), and the average rate of state change (*r*). To the left of each plot, possible starting states are circled, relative rates are shown by arrowhead size. **(a)** *π*_*a*_ = 0.5, *r*_*ab*_ = 1, fully unbiased state change. **(b)** *π*_*a*_ = 0.5, *r*_*ab*_ = 10. **(c)** *π*_*a*_ = 1, *r*_*ab*_ = 1. **(d)** *π*_*a*_ = 1, *r*_*ab*_ = 10. **(e)** *π*_*A*_ = 0.5, *r*_*ab*_ = 1, 50% of *A* sequences are discarded. Note that at low rates (*r* = 10), origination at *A* (**c** and **d**) shows *SP* statistic > 0.5. However, at higher rates (*r* > 10), increased rate of state change from *A* to *B* actually produces lower *SP* statistics. This effect increases as overall rate (*r*) increases. This indicates that the *SP* statistic itself is not a good estimator for the relative rate of state change, and is why we only interpret *p* values resulting from comparison of the *SP* statistic to a null distribution (i.e. the *SP* test).
(PDF)

S6 Fig Simulations across large ladder phylogenies with slow rates of state change. Five randomly chosen phylogenies from simulations using ladder phylogenies of 1000 tips, unbiased rates of state change (*r*_*ab*_ = 1) and *r* = 1 state change/mutation/site. These trees appear as diagonal lines due to their size. For ease of visualization, tips at state *A* (middle) and *B* (left) are shown separately. Single dots usually represent changes along tip branches. For each tree, the observed and permutated *SP* test statistics from *A* to *B* (blue line/green histogram, respectively) and *B* to *A* (red line/orange histogram, respectively) are shown on the right. These confirm that the uncorrected *SP* test has a high rate of false positives across large trees with slow rates of state change.
(PDF)

S7 Fig Tip-to-state change ratio of simulations and empirical BCR datasets. The tip to state change ratio was calculated for each lineage with at least 20 unique sequences in each dataset. For computational efficiency, all lineages were sampled to a maximum tip-to-state change ratio of 500. The top three panels show results from the first 5 repetitions of simulations with large ladder phylogenies used in **Figs 4** and S6. These simulations used unbiased state change (*π*_*a*_ = 0.5, *r*_*ab*_ = 1) and contained 100 trees per repetition. Slow (*r* = 1) rates of state change give high tip to state change ratios, while faster simulations (*r* = 10 or 100) have much lower ratios. The bottom three panels show results from three empirical datasets. The fourth panel from the top is the HIV dataset (**Fig 5**) containing only CD19^hi^ and GCBC cells [6]. The fifth is the isotype dataset (**Fig 6**) [36]. The bottom panel is previously processed data from a recent study containing thymus and blood samples from myasthenia gravis patients [8]. In all three cases, the tip-to-state change ratio of empirical BCR data is more characteristic of simulations in which *r* = 10 changes/mutation/site. The *SP* test performs well under these rates (**Figs 2**,**4 and** S9).
(PDF)

S8 Fig Down-sampling simulation analysis. Simulations are the same as in **Fig 4**, but the proportion of significant *SP* tests from *B* to *A* rather than *A* to *B* are shown. **(a)** Unbiased simulations, significant *SP* test values indicate false positives, as in **Fig 4**. **(b)** Simulations with rates biased from *A* to *B*. As expected, there is no appreciable rate of significant *SP* statistics from *B* to *A* in this scenario.
(PDF)

S9 Fig Repeating previous two-state simulations with down-sampling. *SP* test results performed on the same simulation data as in **Fig 2**, but with each lineage down-sampled to a maximum tip to state change ratio of 20. Results are largely unchanged except for slight reduction in power at higher rates of state change.
(PDF)

S10 Fig *SP* test δ values for differentiation of B cell subtypes within HIV+ subjects. Distribution of *SP* test δ values between all four B cell subtypes included (different colors) for each subject.
(PDF)
