# Supplementary material for: Phylogenetic analysis of migration, differentiation, and class switching in B cells
Source: PLoS Comput Biol. 2022 Apr 25;18(4):e1009885. doi: 10.1371/journal.pcbi.1009885 (PMC9037912; doi:10.1371/journal.pcbi.1009885)
Supplement: S1 Appendix — (PDF) [file pcbi.1009885.s001.pdf]

Appendix S1. Maximum ambiguity resolution of polytomies. B cell lineage trees frequently have clusters of nodes separated by zero-length branches called polytomies. Polytomies represent a high degree of uncertainty in ancestor-descendant relationships within phylogenetic trees (Panel a in **Fig A**) and can be resolved into a multiple distinct, equally supported sets of bifurcating nodes (Panel b in **Fig SA**). We took several steps to ensure that polytomies within each tree were resolved in a manner that appropriately represents this uncertainty. First, all polytomies were re-ordered using nearest-neighbor interchange (NNI) moves to minimize the number of label switches along the tree beginning with the germline sequence and moving down (Panel c in **Fig A**). Then, each polytomy was arranged to a maximum ambiguity configuration, which has the largest number of distinct maximally parsimonious node switching histories (Panel d in **Fig A**). To accomplish this, we used the Sankoff parsimony algorithm to label the state at each internal node of the tree. For each polytomy, we then removed all zero-length branches connecting the ancestral node of the polytomy (*A*; Panel d in **Fig A** node 1) with the descendant nodes of the polytomy (*D*). We then joined all *D* nodes with the same state into separate subtrees, and attached the upper-most nodes of these new single-type subtrees (*T*; Panel d in **Fig A** nodes 3, 4, and 5) to node *A* in a balanced manner. To represent switch types across ambiguous internal node sets, maximum parsimony trajectories were randomly chosen in the backtrace step of the Sankoff algorithm. This process moves down the tree beginning at the root node, and is performed 100 times for each tree. The mean value of each switch type across these repetitions is reported. However, for polytomies with more than two states, there are multiple distinct ways the nodes *T* could be joined to node *A*. To account for this, for each repetition of the sampling procedure, the nodes *T* within each resolved polytomy are randomly swapped using an NNI move. Further, switches were not counted between polytomies and their descendant nodes *D*; rather, the state of each descendant node was randomly chosen from all states contained in the polytomy which could be that node's ancestor with equal parsimony (Panel d in **Fig A** node 6). This latter step effectively allows polytomies to be skipped while maintaining the same number of switches along the tree. This may slightly disfavor switches from states that are ancestral to, but not contained within, a polytomy. These procedures ensured that the minimum number of label switches occurred within each polytomy, and that all distinct sets of label switches along the polytomy are represented. These methods have been implemented in the B cell phylogenetic software package IgPhyML v1.1.1 (bitbucket.org/kleinstein/igphyml). The level of polytomy resolution may be specified while running IgPhyML.

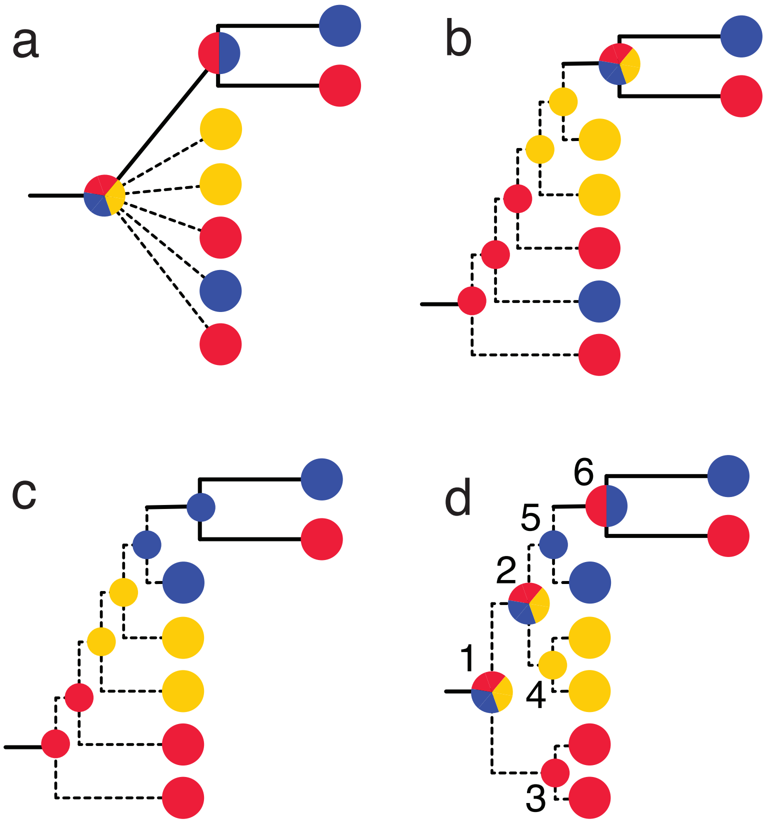

Fig A: Representations of a polytomy. Trait values are represented by red, blue, and yellow colored nodes. Dashed lines represent zero-length branches (no mutations), while solid lines represent nonzero-length branches. Node colors show maximum parsimony predicted trait values. Multi-colored nodes represent multiple equally parsimonious states. **a)** A polytomy represented using a multifurcating node. **b)** Random resolution of the polytomy into a binary tree gives more switches than needed (4), and places red at the root. **c)** Maximum parsimony resolution reduces the total number of switches (3) but, this configuration places the order of switching along the tree as red, yellow, blue, red, when in reality the order of switching cannot be determined. This leads to a switch count of red-yellow=1, yellow-blue=1, blue-red=1. **d)** Maximum parsimony and maximum ambiguity configuration appropriately represents possible switching scenarios under maximum parsimony. The root node (node 1) can be either color with equal parsimony score (3). Switching histories are sampled, starting at node 1 and moving down the tree. Switches are not counted across nonzero branches leading from the polytomy (e.g. between node 5 and node 6). Instead, the state at node 6 is randomly chosen among maximally parsimonious states represented by the polytomy (i.e. red and blue). Further, nodes 3, 4, and 5 are swapped each sampling repetition to ensure each state as equal probability of being at the root node. After multiple sample repetitions, the average number of each type of switch is recorded. The 3 switches that occur are distributed accordingly: red-blue=0.83 switches, blue-red=0.83, red-yellow=0.33, yellow-red=0.33, yellow-blue=0.33, blue-yellow=0.33.
