# Supplementary material for: Phylogenetic analysis of migration, differentiation, and class switching in B cells
Source: PLoS Comput Biol. 2022 Apr 25;18(4):e1009885. doi: 10.1371/journal.pcbi.1009885 (PMC9037912; doi:10.1371/journal.pcbi.1009885)
Supplement: S2 Appendix — (PDF) [file pcbi.1009885.s002.pdf]

Appendix S2. Processing of empirical datasets. Our isotype analysis dataset was aimed at understanding isotype switching patterns in human children, and consisted of BCR mRNA sequences obtained from peripheral blood samples taken from a human child each year from age 1 to 3 years old [1]. Preprocessing was performed with pRESTO v0.5.13 [2]. Quality control was performed by first removing all sequences with a Phred quality score < 20, length < 300bp, or any missing ("N") nucleotides. The 3' and 5' ends of each read were matched to forward and constant region primers with a maximum error rate of 0.1. The region adjacent to the constant region primer was exactly matched to sub-isotype specific internal constant region sequences obtained from [1]. Only sequences with the same isotype predicted by their constant region primer and internal constant region sequence were retained. Identical reads were collapsed and identical sequences observed only once were discarded. V(D)J assignment was performed using IgBLAST v 1.13 [3] against the IMGT human germline reference database (IMGT/GENE-DB v3.1.24; retrieved August 3rd, 2019; [4]). Putatively non-productively rearranged sequences were removed. To infer clonal clusters, sequences were first partitioned based on common IGHV and IGHJ annotation, and junction region length. Within these groups, sequences differing from one another by a normalized Hamming distance of 0.1 within the junction region were clustered into clones using single linkage hierarchical clustering [5]. The V and J genes of unmutated germline ancestors for each clone were then constructed with D segment and N/P regions masked by "N" nucleotides. Clonal clustering and germline sequence reconstruction were performed with Change-O v1.0.0 [6]. Error resulting from repeated sequencing of the same molecule was reduced using a similar approach to [7]. Namely, sequences were removed if they differed by a Hamming distance of 1 from another sequence found 100 times more frequently, or if they differed by Hamming distance of 2 from another sequence found 1000 times more frequently, and so on following a frequency ratio cutoff of $10^{(\text{Hamming distance}+1)}$.

References

1. Nielsen SCA, Roskin KM, Jackson KJL, Joshi SA, Nejad P, Lee J-Y, et al. Shaping of infant B cell receptor repertoires by environmental factors and infectious disease. Science Translational Medicine. 2019;11: eaat2004. doi:10.1126/scitranslmed.aat2004

2. Vander Heiden JA, Yaari G, Uduman M, Stern JNH, O'Connor KC, Hafler DA, et al. pRESTO: a toolkit for processing high-throughput sequencing raw reads of lymphocyte receptor repertoires. Bioinformatics. 2014;30: 1930–1932. doi:10.1093/bioinformatics/btu138

3. Ye J, Ma N, Madden TL, Ostell JM. IgBLAST: an immunoglobulin variable domain sequence analysis tool. Nucl Acids Res. 2013;41: W34–W40. doi:10.1093/nar/gkt382

4. Giudicelli V, Chaume D, Lefranc M-P. IMGT/GENE-DB: a comprehensive database for human and mouse immunoglobulin and T cell receptor genes. Nucl Acids Res. 2005;33: D256–D261. doi:10.1093/nar/gki010

5. Gupta NT, Adams KD, Briggs AW, Timberlake SC, Vigneault F, Kleinstein SH. Hierarchical Clustering Can Identify B Cell Clones with High Confidence in Ig Repertoire Sequencing Data. The Journal of Immunology. 2017;198: 2489–2499. doi:10.4049/jimmunol.1601850

6. Gupta NT, Vander Heiden JA, Uduman M, Gadala-Maria D, Yaari G, Kleinstein SH. Change-O: a toolkit for analyzing large-scale B cell immunoglobulin repertoire sequencing data. Bioinformatics. 2015;31: 3356–3358. doi:10.1093/bioinformatics/btv359

7. Safonova Y, Pevzner PA. IgEvolution: clonal analysis of antibody repertoires. bioRxiv. 2019; 725424. doi:10.1101/725424
