# Supplementary figures and images for: Phylogenetic analysis of migration, differentiation, and class switching in B cells
Source: PLoS Comput Biol. 2022 Apr 25;18(4):e1009885. doi: 10.1371/journal.pcbi.1009885 (PMC9037912; doi:10.1371/journal.pcbi.1009885)

A) Extrafollicular reaction

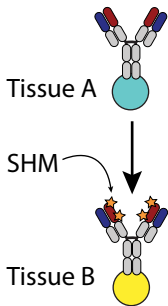

B) GC intermediary

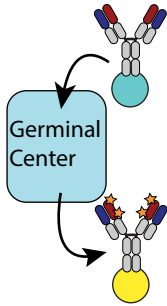

C) GC temporal seeding

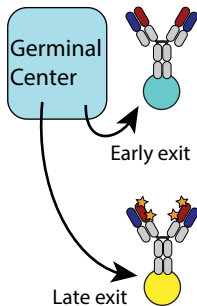

D) Inferred phylogeny

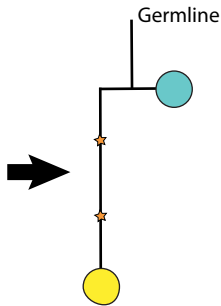

Supplement: S1 Fig — (a) Direct migration from tissue A, followed by extrafollicular mutation in tissue B. (b) Migration from tissue A, through a germinal center where SHM accumulates, and then migration to tissue B. (c) Early germinal center exit to tissue A, late germinal center exit to tissue B. This is not actually migration between tissue A and B. (d) Inferred phylogenetic tree in all three cases showing tissue A as the likely ancestral state compared to tissue B. (PDF) [file pcbi.1009885.s003.pdf]

*PS* test  $p$  value,  $\delta < 0$

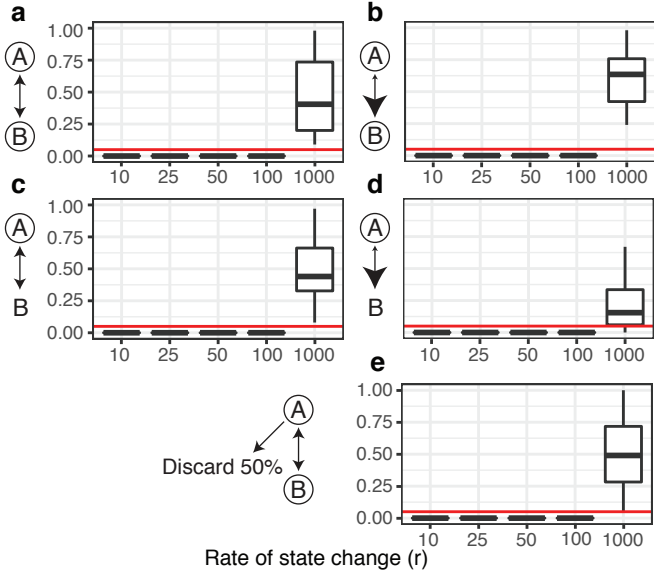

Supplement: S3 Fig — Distribution of PS test p values for the hypothesis that δ < 0 from two state simulation analyses. See Fig 2 for analysis of the same data with the SP test. In these simulations, change between state A and B was determined by the probability of starting in A (πa), relative rate of migrating from A to B (rab), and the average rate of state change (r). To the left of each plot, possible starting states are circled, relative rates are shown by arrowhead size. (a) πa = 0.5, rab = 1, fully unbiased state change. (b) πa = 0.5, rab = 10. (c) πa = 1, rab = 1. (d) πa = 1, and rab = 10. (e) πA = 0.5, rab = 1, 50% of A sequences are discarded. Red lines show the cutoff of p value = 0.05. (PDF) [file pcbi.1009885.s005.pdf]

SC test  $p$  value,  $\delta > 0$

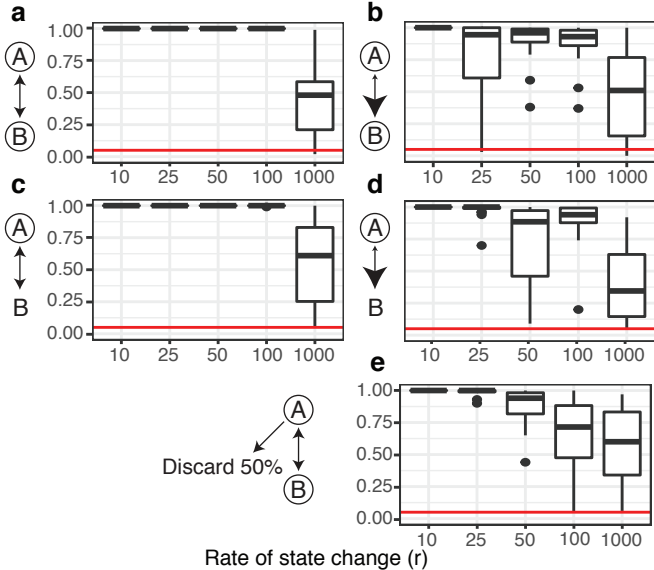

Supplement: S4 Fig — Distribution of SC test p values for the hypothesis that δ > 0 from two state simulation analyses. See Fig 2 for analysis of the same data with the SP test. In these simulations, change between state A and B was determined by the probability of starting in A (πa), relative rate of migrating from A to B (rab), and the average rate of state change (r). To the left of each plot, possible starting states are circled, relative rates are shown by arrowhead size. (a) πa = 0.5, rab = 1, fully unbiased state change. (b) πa = 0.5, rab = 10. (c) πa = 1, rab = 1. (d) πa = 1, and rab = 10. (e) πA = 0.5, rab = 1, 50% of A sequences are discarded. Red lines show the cutoff of p value = 0.05. (PDF) [file pcbi.1009885.s006.pdf]

SP statistic, A to B

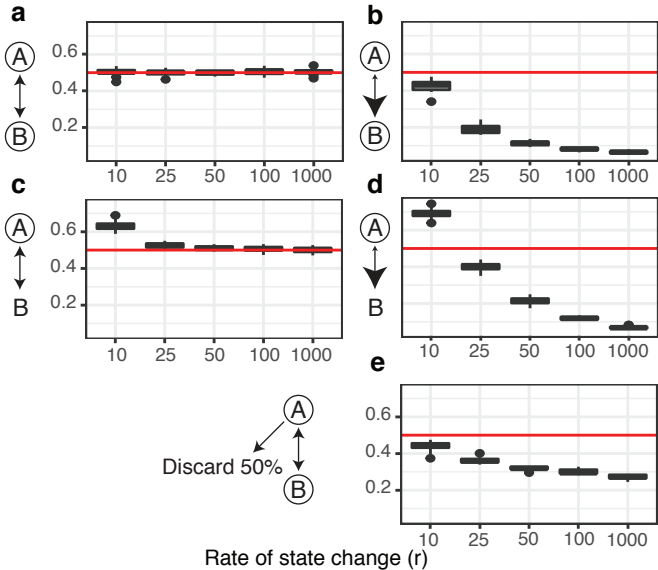

Supplement: S5 Fig — Distribution of raw SP statistics from A to B in two state simulations. The red line is at 0.5, showing equal switch frequency. See Fig 2 for analysis of the same data with the full SP test. In these simulations, change between state A and B was determined by the probability of starting in A (πa), relative rate of migrating from A to B (rab), and the average rate of state change (r). To the left of each plot, possible starting states are circled, relative rates are shown by arrowhead size. (a) πa = 0.5, rab = 1, fully unbiased state change. (b) πa = 0.5, rab = 10. (c) πa = 1, rab = 1. (d) πa = 1, rab = 10. (e) πA = 0.5, rab = 1, 50% of A sequences are discarded. Note that at low rates (r = 10), origination at A (c and d) shows SP statistic > 0.5. However, at higher rates (r > 10), increased rate of state change from A to B actually produces lower SP statistics. This effect increases as overall rate (r) increases. This indicates that the SP statistic itself is not a good estimator for the relative rate of state change, and is why we only interpret p values resulting from comparison of the SP statistic to a null distribution (i.e. the SP test). (PDF) [file pcbi.1009885.s007.pdf]

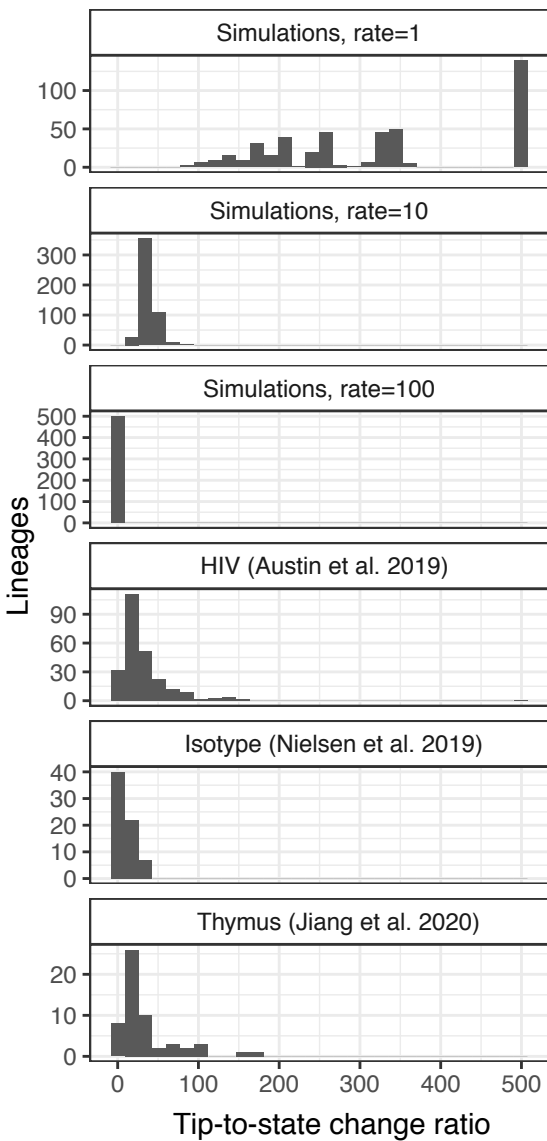

Supplement: S7 Fig — The tip to state change ratio was calculated for each lineage with at least 20 unique sequences in each dataset. For computational efficiency, all lineages were sampled to a maximum tip-to-state change ratio of 500. The top three panels show results from the first 5 repetitions of simulations with large ladder phylogenies used in Figs 4 and S6. These simulations used unbiased state change (πa = 0.5, rab = 1) and contained 100 trees per repetition. Slow (r = 1) rates of state change give high tip to state change ratios, while faster simulations (r = 10 or 100) have much lower ratios. The bottom three panels show results from three empirical datasets. The fourth panel from the top is the HIV dataset (Fig 5) containing only CD19hi and GCBC cells [6]. The fifth is the isotype dataset (Fig 6) [36]. The bottom panel is previously processed data from a recent study containing thymus and blood samples from myasthenia gravis patients [8]. In all three cases, the tip-to-state change ratio of empirical BCR data is more characteristic of simulations in which r = 10 changes/mutation/site. The SP test performs well under these rates (Figs 2,4 and S9). (PDF) [file pcbi.1009885.s009.pdf]

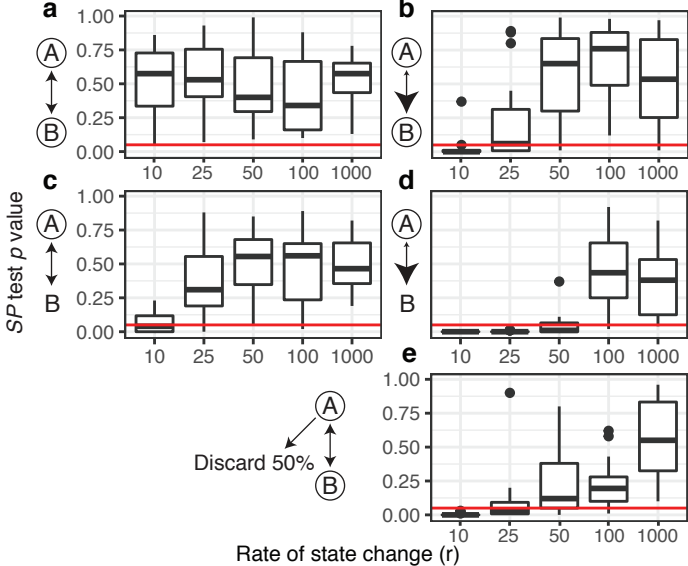

Supplement: S9 Fig — SP test results performed on the same simulation data as in Fig 2, but with each lineage down-sampled to a maximum tip to state change ratio of 20. Results are largely unchanged except for slight reduction in power at higher rates of state change. (PDF) [file pcbi.1009885.s011.pdf]

Bootstrap replicates

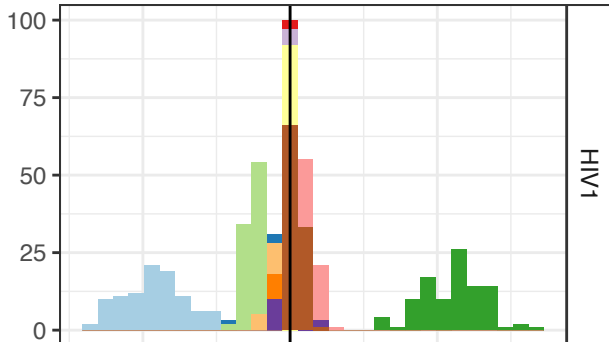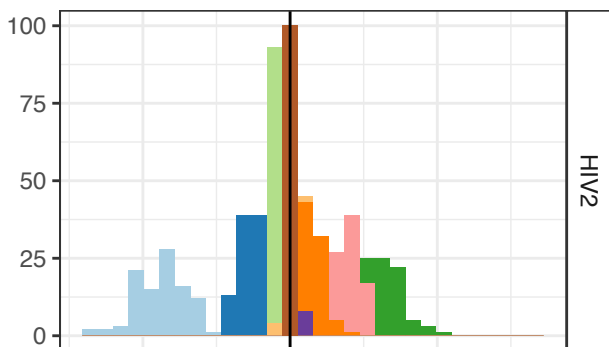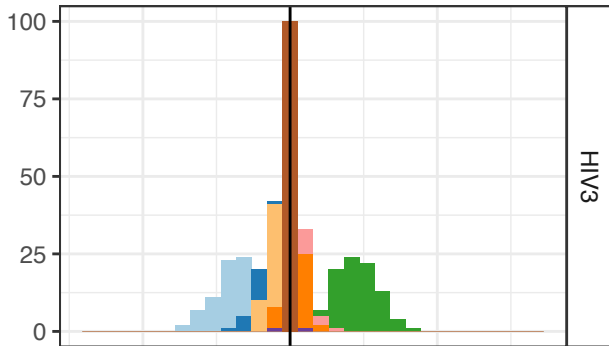

From:To

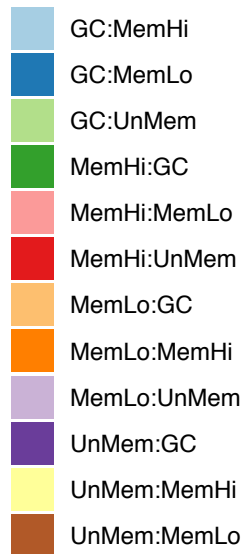

Supplement: S10 Fig — Distribution of SP test δ values between all four B cell subtypes included (different colors) for each subject. (PDF) [file pcbi.1009885.s012.pdf]
