# Supplementary material for: Phylogenetic analysis of migration, differentiation, and class switching in B cells
Source: PLoS Comput Biol. 2022 Apr 25;18(4):e1009885. doi: 10.1371/journal.pcbi.1009885 (PMC9037912; doi:10.1371/journal.pcbi.1009885)
Supplement: S2 Fig — (PDF) [file pcbi.1009885.s004.pdf]

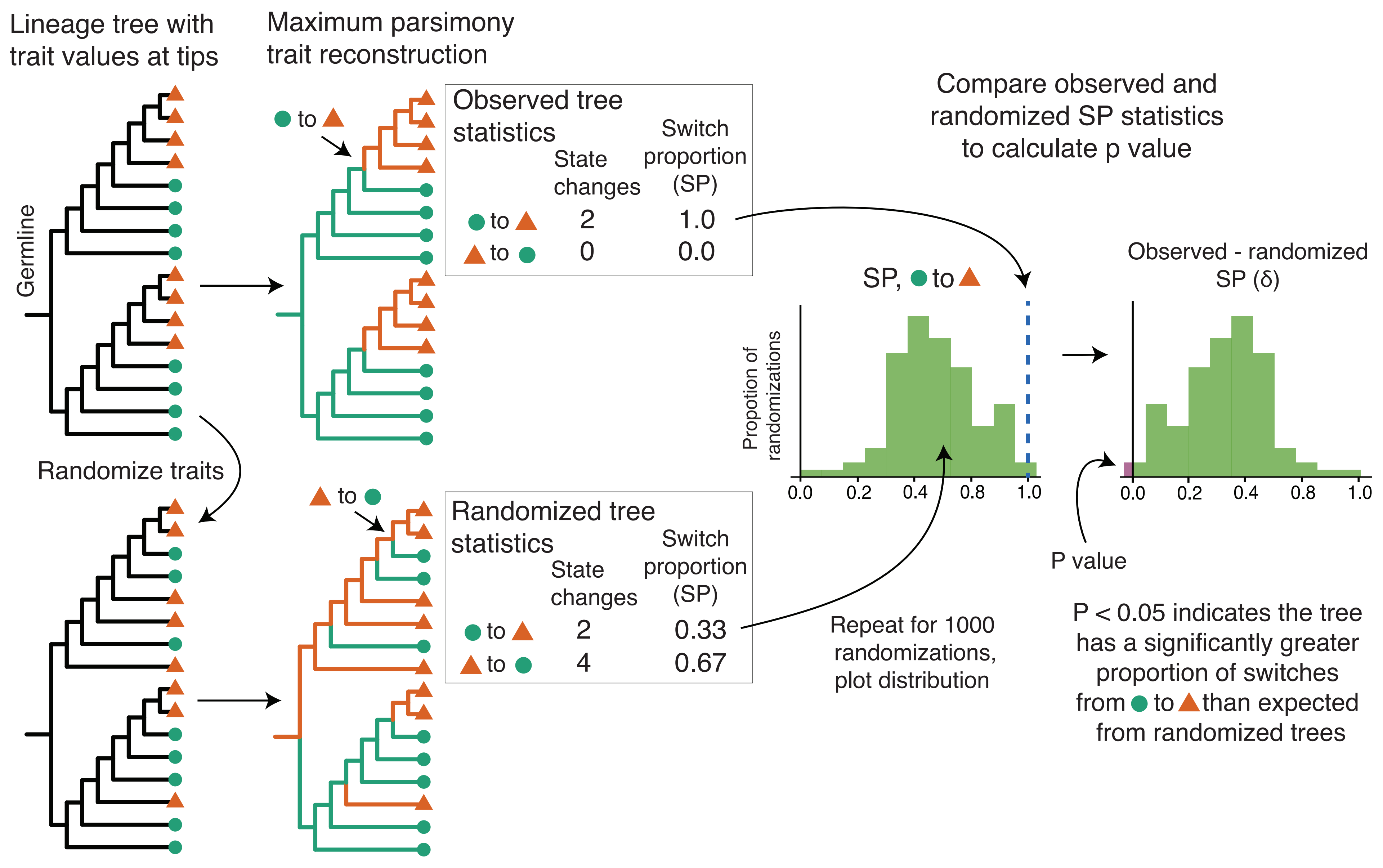

Lineage tree with trait values at tips
Maximum parsimony trait reconstruction
Germline
● to ▲
Observed tree statistics
State changes
Switch proportion (SP)
● to ▲ 2 1.0
▲ to ● 0 0.0
Randomize traits
▲ to ●
Randomized tree statistics
State changes
Switch proportion (SP)
● to ▲ 2 0.33
▲ to ● 4 0.67
Compare observed and randomized SP statistics to calculate p value
SP, ● to ▲
Propotion of randomizations
0.0 0.2 0.4 0.8 1.0
Observed - randomized SP (δ)
0.0 0.2 0.4 0.8 1.0
Repeat for 1000 randomizations, plot distribution
P value
P < 0.05 indicates the tree has a significantly greater proportion of switches from ● to ▲ than expected from randomized trees
