# Supplementary material for: Phylogenetic analysis of migration, differentiation, and class switching in B cells
Source: PLoS Comput Biol. 2022 Apr 25;18(4):e1009885. doi: 10.1371/journal.pcbi.1009885 (PMC9037912; doi:10.1371/journal.pcbi.1009885)
Supplement: S6 Fig — Five randomly chosen phylogenies from simulations using ladder phylogenies of 1000 tips, unbiased rates of state change (rab = 1) and r = 1 state change/mutation/site. These trees appear as diagonal lines due to their size. For ease of visualization, tips at state A (middle) and B (left) are shown separately. Single dots usually represent changes along tip branches. For each tree, the observed and permutated SP test statistics from A to B (blue line/green histogram, respectively) and B to A (red line/orange histogram, respectively) are shown on the right. These confirm that the uncorrected SP test has a high rate of false positives across large trees with slow rates of state change. (PDF) [file pcbi.1009885.s008.pdf]

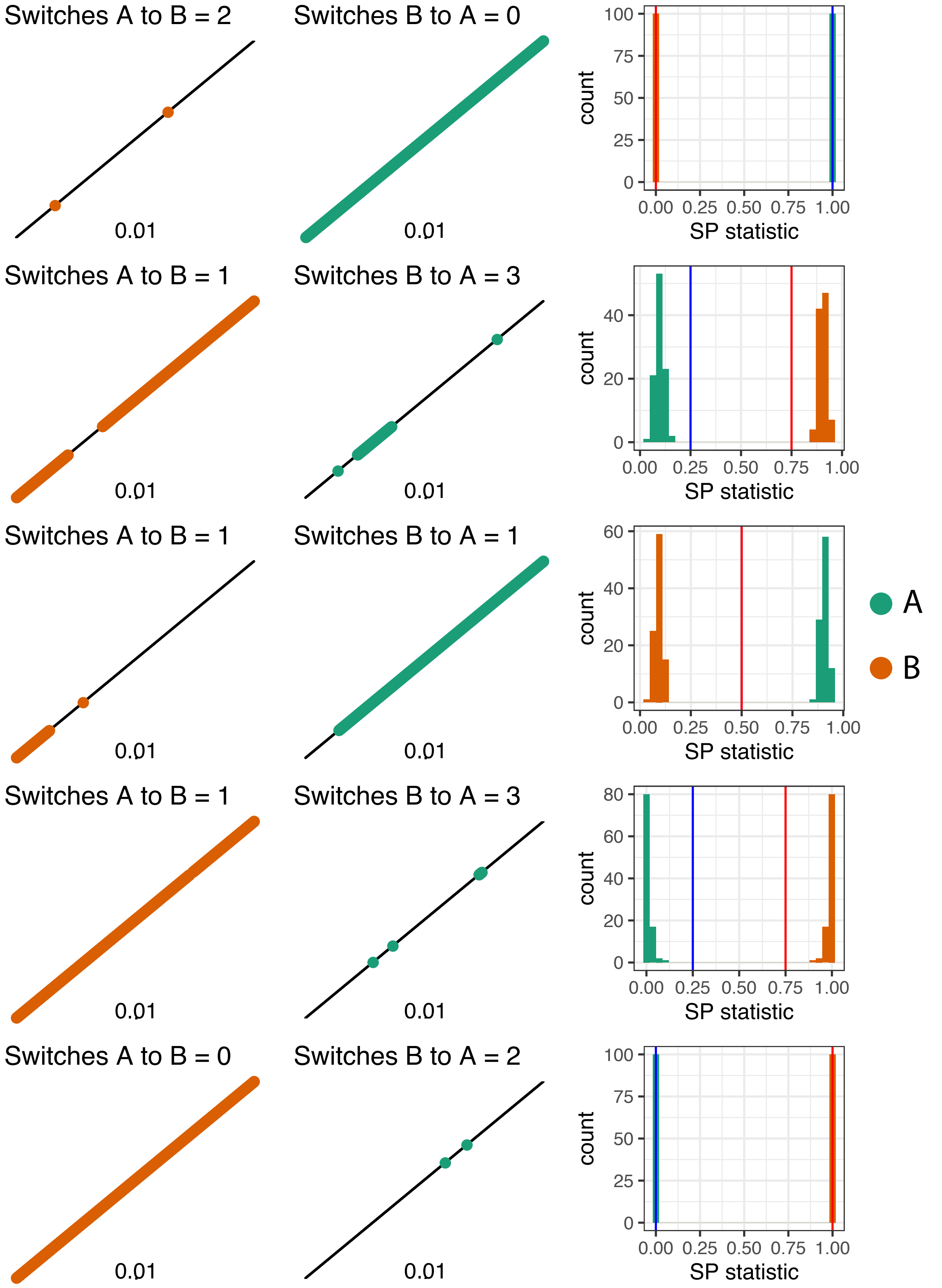

Switches A to B = 2
Switches B to A = 0
0.01
0.01
count
SP statistic
Switches A to B = 1
Switches B to A = 3
Switches A to B = 1
Switches B to A = 1
Switches A to B = 1
Switches B to A = 3
Switches A to B = 0
Switches B to A = 2
A
B
0.00 0.25 0.50 0.75 1.00
