# Supplementary material for: Phylogenetic analysis of migration, differentiation, and class switching in B cells
Source: PLoS Comput Biol. 2022 Apr 25;18(4):e1009885. doi: 10.1371/journal.pcbi.1009885 (PMC9037912; doi:10.1371/journal.pcbi.1009885)
Supplement: S8 Fig — Simulations are the same as in Fig 4, but the proportion of significant SP tests from B to A rather than A to B are shown. (a) Unbiased simulations, significant SP test values indicate false positives, as in Fig 4. (b) Simulations with rates biased from A to B. As expected, there is no appreciable rate of significant SP statistics from B to A in this scenario. (PDF) [file pcbi.1009885.s010.pdf]

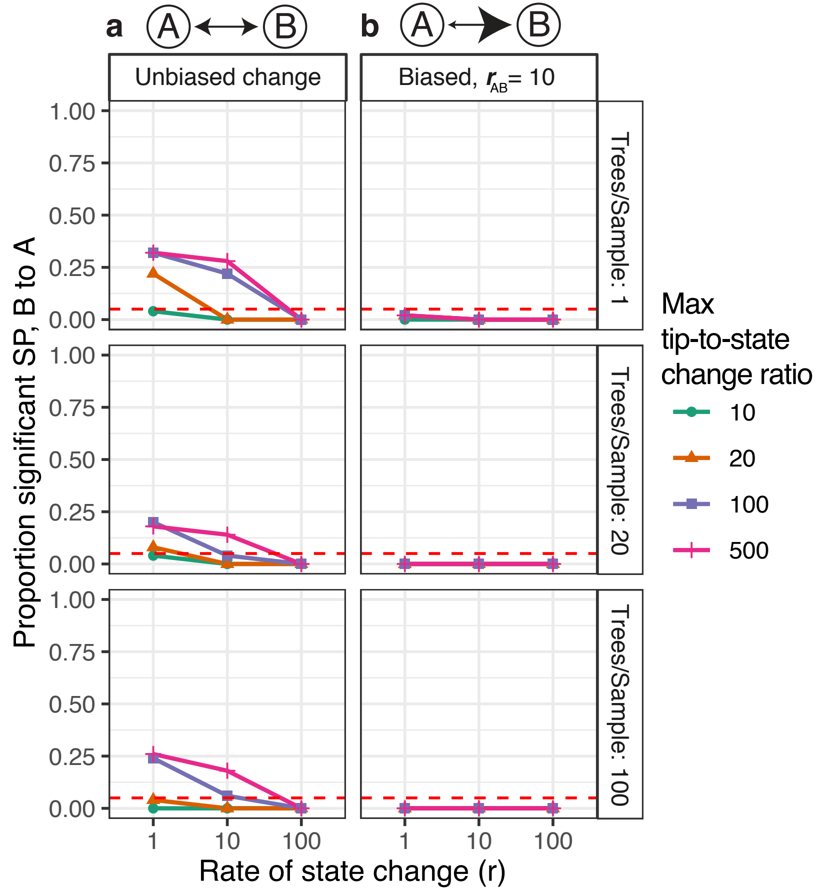

a
A
B
b
A
B
Unbiased change
Biased, r_{AB}= 10
Trees/Sample: 1
Trees/Sample: 20
Trees/Sample: 100
Proportion significant SP, B to A
1.00
0.75
0.50
0.25
0.00
1
10
100
Rate of state change (r)
Max tip-to-state change ratio
10
20
100
500
